# ‘Wear advantage’ of mobile‐bearing unicompartmental knee arthroplasty is a myth: Higher volumetric wear without reduced revision rates compared to the fixed‐bearing design: A systematic review and meta‐analysis

**DOI:** 10.1002/jeo2.70837

**Published:** 2026-07-09

**Authors:** Oliver G. B. Dixon, Emily M. London, Ahmed Elsaifi, Jacobus H. Müller, Nick J. London

**Affiliations:** ^1^ York and Scarborough Teaching Hospitals NHS Foundation Trust York UK; ^2^ Mid Yorkshire Teaching NHS Trust Wakefield UK; ^3^ ReSurg, SA Nyon Switzerland; ^4^ Carnegie School of Sport Leeds Beckett University Leeds UK; ^5^ Harrogate and District NHS Foundation Trust Harrogate UK; ^6^ Yorkshire Knee Clinic Yorkshire UK

**Keywords:** fixed‐bearing, knee, mobile‐bearing, unicompartmental, wear

## Abstract

**Purpose:**

This systematic review and meta‐analysis aims to comprehensively compare wear between mobile‐ and fixed‐bearing unicompartmental knee arthroplasty (UKA) using clinical, in vitro and registry data.

**Methods:**

This study was conducted in accordance with Preferred Reporting Items for Systematic Reviews and Meta‐Analyses guidelines and registered with PROSPERO. PubMed, EMBASE and Epistemonikos were searched. Clinical, registry, in vitro and in silico studies reporting wear outcomes in mobile‐ and fixed‐bearing UKA were included. Risk of bias was assessed using the mixed methods appraisal tool (MMAT), and pooled analyses were performed using mixed‐effects models.

**Results:**

The electronic literature search identified 5688 studies, of which 64 met the inclusion criteria after screening. For UKAs implanted after 2000, there were no statistically significant differences in clinical revision rates for wear between mobile‐ and fixed‐bearing designs, either overall (<1% vs. 1%; *p* = n.s.) or at a follow‐up of >10 years (2% vs. 1%; *p* = n.s.). A statistically significant difference in in vitro volumetric wear was observed (*p* < 0.001), with mobile‐bearing constructs demonstrating a higher wear rate (5.54 mm^3^/MC) compared to fixed‐bearing constructs (1.69 mm^3^/MC).

**Conclusion:**

This comprehensive review of all available data demonstrates that fixed‐ and mobile‐bearing medial UKAs show no difference in the clinical incidence of revision for wear but reveals a statistically significant advantage for fixed‐bearing medial UKAs regarding volumetric wear, consistent with the original hypothesis of our study. Fixed‐bearing implants show more uniform wear patterns, suggesting potentially more predictable long‐term performance.

**Level of Evidence:**

Level IV.

## Graphical abstract

Mobile‐ vs Fixed‐Bearing Unicompartmental Knee Arthroplasty.

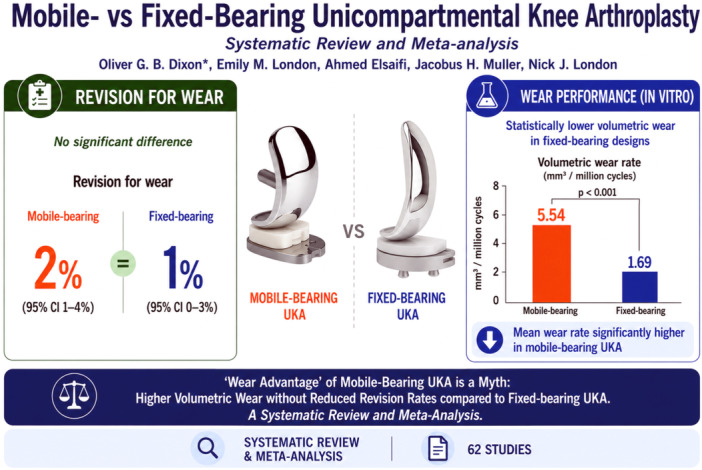

**Abbreviations**
C.O.I.: conflict of interest
MC: million cycles
MMAT: mixed methods appraisal tool
NJR: National Joint Registry
n.r.: not reported
n.s.: not significant
PRISMA: Preferred Reporting Items for Systematic Reviews and Meta‐Analyses
TKA: total knee arthroplasty
UKA: unicompartmental knee arthroplasty

## INTRODUCTION

While the benefits of unicompartmental knee arthroplasty (UKA) are recognised, controversy persists regarding the optimal bearing surface design for UKA implants and whether mobile‐ or fixed‐bearing implants offer the best results in terms of implant longevity. Fixed‐bearing designs use a flat polyethene bearing surface that locks onto the tibial component, whereas mobile‐bearing designs allow for rotation and translation relative to the tibial baseplate [35]. The theory behind the use of the mobile‐bearing implant is that it reduces the rate of polyethylene wear, debris‐induced osteolysis and subsequent loosening of the joint replacement components. However, dislocation is a recognised complication associated with the use of the mobile‐bearing implant [7]. In contrast, fixed‐bearing designs reduce backside wear and are at negligible risk of dislocation.

Following the introduction of UKA, mobile‐bearing implants initially predominated in the United Kingdom; however, according to the most recent National Joint Registry (NJR) report, fixed‐bearing designs are now the most commonly used [2]. The widespread adoption of mobile‐bearing implants was largely justified by their purported reduction in polyethylene wear, based on evidence from studies published approximately 25 years ago. More recent systematic reviews have failed to demonstrate clear superiority [63]. Around the year 2000, the use of vacuum irradiation for high molecular weight polyethylene to reduce the amount of oxidation, free radical formation, and resultant wear of the liner came into use for UKA. This coincided with the use of cross‐linked polyethylene in UKA, which may also affect previously reported wear rates. In contrast to historic clinical studies, in vitro studies have reported higher wear rates with mobile‐bearing designs as opposed to fixed‐bearing implants [14]. The latest data from the UK NJR review reported that all‐cause revisions for mobile UKA were 12.2 per 1000 prosthesis‐years in comparison to 6.7 for implants incorporating a fixed‐bearing design [2]. While this difference may be influenced by the surgeon's experience with the mobile design, it may also be due to the rate of wear. In addition to this, the UK NJR reported a rate of revision due to wear of only 0.65 per 1000 prosthesis‐years for fixed‐bearing cemented designs in comparison to a rate of 1.46 for mobile‐bearing designs [2]. While there are numerous reasons for revision surgery and implant wear, these observed differences may be influenced by the type of bearing design.

Most published systematic reviews comparing fixed‐ and mobile‐bearing UKA are based on clinical studies [18, 43, 46, 57, 76, 77], with only a minority incorporating registry data [1], and no analysis including in vitro or in vivo evidence. Given the reported differences and conflicting evidence in the available literature to date, this systematic review and meta‐analysis aims to evaluate all studies (clinical, in vitro and registry databases) to provide the best possible evidence on the wear differences between the two implant designs. The hypothesis is that there is no significant difference in terms of wear between the two implant designs.

## MATERIALS AND METHODS

### Search strategy

This systematic review with meta‐analysis was performed in accordance with the Preferred Reporting Items for Systematic Reviews and Meta‐Analyses criteria (PRISMA). The protocol, which describes the search strategy, inclusion criteria, risk of bias assessment, and data synthesis plan, was submitted to PROSPERO on 21 March 2025 and registered on 9 April 2025 (Reference: CRD420251003417). The electronic search was conducted on PubMed, EMBASE and Epsitemonikos between March 2000 and March 25, 2025 (see Supporting Information S1).

### Inclusion and exclusion criteria

The authors sought to include reports on failure due to wear (incidence of wear) in mobile‐bearing or fixed‐bearing UKA from clinical studies on adult patients treated for primary medial compartment osteoarthritis. In addition, the authors also sought to include reports on linear or volumetric wear from laboratory tests or in silico investigations. Studies were excluded if patients had a previous or concurrent lateral UKA, high tibial osteotomy or patellofemoral arthroplasty; had conditions other than primary osteoarthritis; or received individualised UKA implants. The authors included randomised controlled trials; comparative or single‐arm prospective, ambispective, or retrospective studies reporting ≥5‐year survival; registry‐based studies reporting ≥5‐year survival; and in vitro or in silico studies reporting wear, published in the past 15 years. Reviews (including narrative, scoping, systematic, meta‐analyses, or network meta‐analyses), case reports or small series (*n* < 10), technical notes, letters to the editor, editorials or corrections were excluded. The authors also did not consider records in languages other than English or French to avoid translation errors.

### Screening and study selection

Two authors independently screened the titles and abstracts of all records identified during the electronic database search using a web‐based tool (Rayyan [49]). Disagreements between the two authors on the eligibility of a record were resolved through review and discussion. Next, the full texts of papers that passed the title and abstract screening were retrieved and individually screened by the same two authors. Disagreements between the two authors on the eligibility of a record were resolved through review and discussion. The reference lists of all records that passed full‐text screening were examined for relevant studies that might have been missed during the formal search and screening. Additionally, studies citing these records were reviewed for possible inclusion.

### Risk of bias and publication bias assessment

Three authors independently assessed the methodological quality of the included studies using the 2018 version of the mixed methods appraisal tool (MMAT) [24, 50, 52]. The MMAT is a critical appraisal tool for systematic mixed studies reviews, allowing assessment of the methodological quality of qualitative, quantitative and mixed‐methods studies, including randomised and non‐randomised designs, quantitative descriptive studies and mixed‐methods studies. Disagreements between authors on the methodological quality of a record were resolved through review and discussion. Potential publication bias was assessed using both graphical and statistical methods for subgroups with >10 studies per arm. Visual inspection of funnel plots was performed using logit‐transformed proportions to account for the bounded nature of the data and to minimise artificial asymmetry. Statistical asymmetry was quantified using Peters' linear regression test, which is specifically recommended for meta‐analyses of single‐arm proportions as it maintains a lower type I error rate compared to Egger's test when dealing with binary outcomes. A *p* value < 0.10 was considered indicative of significant funnel plot asymmetry.

### Data extraction

Three authors independently extracted data from the included records for tabulation on a spreadsheet. First, each author extracted the study characteristics, which included the first author, year of publication, journal, study design and whether the authors declared receiving funding or having conflicts of interest (COI). Second, cohort or sample characteristics were extracted, which included the UKA bearing design, number of knees, patients and/or implant samples, age, BMI and indications. Third, variables related to bearing wear were extracted, including reported incidence or rates of wear (number and proportion), quantification of wear‐related damage (damage scores), as well as volumetric (mm^3^/million cycles [MC]), gravimetric (mg/MC) or linear wear (mm/MC).

### Data synthesis and statistical analysis

The authors also pooled the absolute incidence of wear from studies on mobile‐bearing and fixed‐bearing designs. The pooled proportions were estimated using logit transformations with generalised linear mixed models. A subgroup analysis was performed by polyethylene type, classifying UKAs conducted before 2000 as conventional polyethylene and those from 2000 onward as cross‐linked polyethylene. An additional subgroup analysis was performed on cross‐linked polyethylene inserts by comparing the incidence of wear between implants with less than 10 years of follow‐up to implants with more than 10 years of follow‐up. The authors separately pooled volumetric and gravimetric wear rates from in vitro and retrieval studies to estimate the pooled mean for mobile‐ and fixed bearing designs. A common‐effects model was used when heterogeneity was low (*I* ^2^ < 50%) or a random‐effects model when heterogeneity was higher (*I* ^2^ ≥ 50%). Leave‐one‐out sensitivity analyses were performed to evaluate the stability of the meta‐analysis model and identify studies that contribute most to heterogeneity. Statistical analyses were performed using R Studio 2026.01.0 Build 392 (Posit Software, PBC) and R version 4.5.2 (R: A Language and Environment for Statistical Computing. R Foundation for Statistical Computing). A *p* value of <0.05 was deemed statistically significant.

## RESULTS

### Study characteristics and risk of bias assessment

**Figure 1:**
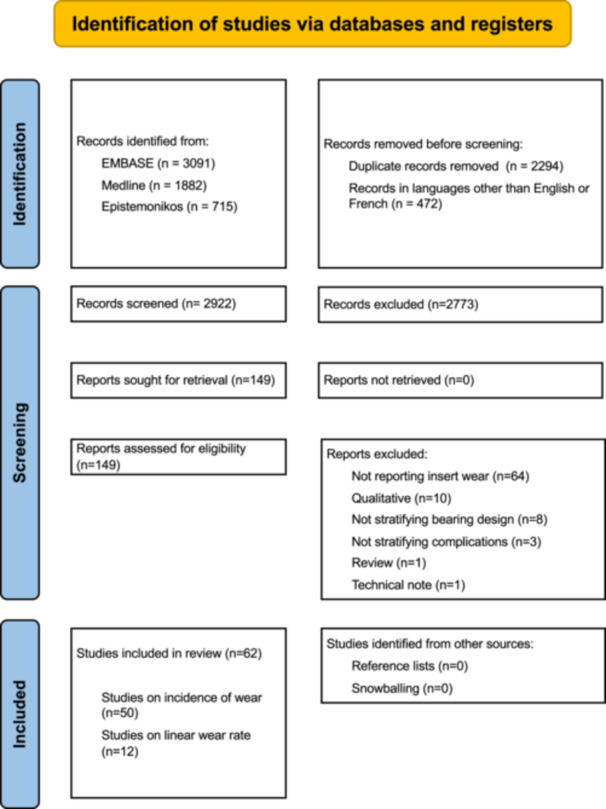
PRISMA flowchart. PRISMA, Preferred Reporting Items for Systematic Reviews and Meta‐Analyses.

The electronic literature search returned 5688 records. Following title, abstract and full‐text screening, 62 studies remained eligible for data extraction (Figure 1) [3, 13, 15, 17, 19, 23, 25, 34, 36, 42, 44, 45, 47, 48, 51, 53, 56, 58, 62, 64, 75]. The 62 eligible studies were published between 2010 and 2025, conducted across multiple countries, and included over 145,000 knees, with individual study sample sizes ranging from 2 to 20,208 knees. Among these studies, 50 reported on the incidence of wear (7 comparative [13, 28, 51, 60, 61, 67, 72] and 43 single‐arm studies [3, 8, 10, 12, 16, 17, 23, 26, 27, 29, 33, 34, 38, 42, 45, 47, 53, 55, 56, 58, 62, 64, 65, 68, 71, 73]) and 12 on wear rate (6 comparative [11, 15, 25, 37, 48, 66] and 6 single‐arm studies [22, 30, 36, 44, 54, 59]) (Tables 1a, 1b, 2a and 2b).

**Table 1a:** Characteristics of comparative studies that report on the incidence of wear between fixed‐ and mobile‐bearing designs.

Author; year	Country	Declarations		Inclusion period	Bearing design	Patients | knees	FU (years)	Incidence of wear	
		Funding	COIs					*n*	Denominator
Registry‐based studies (LoE III comparative; *n* = 2)									
[13]	The Netherlands	None	Relevant	2007–2018	Mobile (cem)	11,017 | 11,025	Med 4.8	15	11,025
					Mobile (uncem)	6930 | 6942	Med 2.0	11	6942
					Fixed (cem)	3639 | 3643	Med 4.1	4	3643
[28]	Australia	n.r.	Relevant	1999–2018	Mobile	– | 20,208	18	37	20,208
					Fixed (molded)	– | 889	15	6	889
					Fixed (AP)	– | 11,461	18	24	11,461
					Fixed (hybrid)	– | 17,822	18	33	17,822
Retrospective (LoE III comparative; *n* = 6)									
[67]	New Zealand	None	Relevant	2000–2017	Mobile (cem)	413 | 496	11.3	3	496
					Mobile (uncem)	867 | 1069	6.8	0	1069
					Fixed	399 | 450	7	0	450
[61]	Italy	n.r.	None	2004–2010	Mobile	67 | –	11.5	0	67
					Fixed (AP)	83 | –	11.5	2	83
[60]	Korea	None	None	2002–2009	Mobile	– | 36	>8	2	36
					Fixed	– | 60	>8	2	60
[51]	France	n.r.	Relevant	1989–1992	Mobile	– | 77	17.2	0	77
					Fixed	– | 79	>15	4	79
[72]	Canada	n.r.	None	1993–2007	Mobile	– | 75	3.6	0	75
				1990–2007	Fixed	– | 110	8.1	7	110

Abbreviations: AP, all‐poly; cem, cemented; COI, conflict of interest; FU, follow‐up; LoE, level of evidence; med, median; n.r., not reported; uncem, uncemented.

**Table 1b:** Characteristics of single‐arm studies that report on the incidence of wear for fixed‐ and mobile‐bearing designs.

Author; year	Country	Declarations		Inclusion period	Bearing design	Patients | knees	FU (years)	Incidence of wear	
		Funding	COIs					*n*	Denominator
Registry‐based studies (LoE IV non‐comparative; *n* = 5)									
[68]	The Netherlands	None	Relevant	2014–2022	Mobile (cem)	– | 8022	2.5	9	8022
					Mobile (uncem)	– | 17,740	2.5	17	17,740
[62]	Norway	n.r.	None	2012–2021	Mobile (cem)	– | 4715	5	4	4715
					Mobile (uncem)	– | 1821	5	7	1821
					Mobile (3rd gen)	– | 908	5	3	908
[69]	The Netherlands	None	Relevant	2007–2019	Mobile (3rd gen)	– | 12,240	5	30	12,240
[45]	UK	None	Relevant	2005–2016	Mobile (cem)	– | 7407	10	7	7407
					Mobile (uncem)	– | 7407	10	7	7407
[6]	Norway	None	n.r.	1999–2012	Mobile (3rd gen)		13	178	4460
Prospective (LoE IV single series; *n* = 8)									
[39]	Singapore	None	None	2003–2007	Fixed	83 | –	>15	3	83
[8]	USA	Relevant	Relevant	2009–2011	Fixed	366 | 411	10.1	1	411
[53]	UK	Relevant	None	1974–1994	Fixed	385 | 479	13.3	10	479
[12]	UK	None	Relevant	2002–2007	Fixed (AP)	– | 92	10	3	214
[41]	Singapore	None	None	2003–2005	Fixed	216 | 263	>10	1	263
[73]	Singapore	None	None	2003–2007	Fixed		>10	1	184
[64]	Korea	n.r.	None	2003–2005	Fixed	64 | 68	8.9	1	68
[42]	France	n.r.	n.r.	1988–1994	Mobile (2nd gen)	40 | 43	10.5	4	43
Retrospective (LoE IV single series; *n* = 31)									
[4]	Italy	None	None	2003–2006	Fixed (AP and m‐b)	52 | 52	18	1	52
[26]	Taiwan	Irrelevant	None	2003–2014	Fixed	292 | 337	14	7	337
[29]	UK	n.r.	None	2008–2010	Mobile		12	6	257
[10]	USA	Relevant	None	2002–2020	Fixed		>5	1	308
[16]	USA	n.r.	Relevant	2000–2009	Fixed	68 | 77	11.2	1	95
[27]	Korea	n.r.	None	2004–2010	Fixed	67 | 67	>8	1	67
[55]	Saudi Arabia	None	None	1988–2009	Fixed	– | 205	>5	6	205
[7]	Korea	None	None	2002–2016	Mobile (3rd gen)		>0.5	1	1853
[40]	Singapore	n.r.	None	2004–2007	Fixed	84 | Propensity (71)	>10	1	71
[65]	Korea	None	None	1992–1997	Fixed	– | 50	12	4	50
[71]	UK	None	None	2001–2010	Fixed		5	1	175
[3]	USA	Relevant	Relevant	2004–2006	Mobile (3rd gen)	577 | –	7.6	2	825
[34]	Korea	n.r.	None	2002–2003	Mobile (3rd gen)	80 | 106	>10	2	106
[19]	Republic of Korea	n.r.	None	2002–2005	Mobile (3rd gen)	147 | 164	>10	11	188
[74]	China	Irrelevant	n.r.	2004–2010	Mobile (cem)		7.2	1	67
[75]	China	Irrelevant	None	2005–2014	Mobile (3rd gen)	634 | 708	6.2	1	708
[20]	USA	Irrelevant	Relevant	2004–2006	Mobile (3rd gen)	143 | 168	10	1	213
[33]	Korea	n.r.	None	2002–2014	Mobile		7.1	3	1410
[70]	UK	None	None	2001–2010	Fixed		5.6	1	175
[31]	Korea	n.r.	None	2002	Mobile (3rd gen)	128 | 166	>10	1	166
[38]	France	n.r.	None	1996–2000	Fixed	64 | 65	>10	4	65
[47]	Brazil	n.r.	None	1990–2013	Fixed (cem)	26 | 27	n.r.	1	17
[5]	France	None	Relevant	1989–1997	Fixed	62 | 70	20	2	160
[17]	France	n.r.	n.r.	1990–2004	Fixed	185 | 212	11.6	1	212
[56]	UK	None	None	1990–2003	Fixed	52 | 56	10.7	2	56
[58]	USA	n.r.	Relevant	2005–2008	Mobile		3.6	1	83
[23]	Germany	Irrelevant	Relevant	1993–2004	Fixed (AP)	– | 88	10.8	–	–
					Fixed (cem)	– | 30		1	30
					Fixed (uncem)	– | 55		3	55
[32]	Korea	No	No	2002–2004	Mobile (3rd gen)	194 | 246	>5	1	246
[9]	Australia	None	No	2000–2004	Fixed	– | 118	5.7	1	118
[21]	France	No	Relevant	1989–2006	Fixed	62 | 65	11.2	3	65

Abbreviations: AP, all‐polyethylene tibial component; cem, cemented; COI, conflict of interest; FU, follow‐up; gen, generation; LoE, level of evidence; m‐b, metal‐backed tibial component; n.r., not reported; uncem, uncemented.

**Table 2a:** Characteristics of comparative studies that report on wear rate between fixed‐ and mobile‐bearing designs.

Author; year	Country	Declarations		Inclusion period	Bearing design	Patients | knees	FU (years)	Wear rate	Comment	Unit
		Funding	COIs							
RCT (LoE I; *n* = 1)										
[25]	Denmark	Relevant	None	2009–2014	Mobile (cem)	48 | 48	5	0.04 (95% CI: 0.02–0.07)		mm/year
					Mobile (uncem)	23 | 23	5	0.05 (95% CI: 0.02–0.08)		mm/year
Knee simulator (LoE V comparative; *n* = 5)										
[48]	USA		Relevant	≥2000	Mobile	– | 3	n.a.	18.35	ISO 14243‐3	mg/MC
					Fixed	– | 3		3.89	ISO 14243‐3	mg/MC
[15]	UK	Relevant	Relevant	≥2000	Mobile	– | 6	n.a.	7.08 ± 1.47	Intermediate kinematics	mm^3^/MC
								3.77 ± 0.76	High kinematics	
								5.54 ± 1.74	Femoral condylar lift off	
					Fixed	– | 6		0.92 ± 0.35	Intermediate kinematics	mm^3^/MC
								1.78 ± 0.60	High kinematics	
								1.78 ± 0.60	Femoral condylar lift off	
[11]	UK	n.r.	n.r.	≥2000	Mobile	– | 3	n.a.	5.72 ± 5.98	Intermediate kinematics	mm^3^/MC
								7.44 ± 4.16	High kinematics	
					Fixed	– | 3		1.99 ± 0.80	Intermediate kinematics	mm^3^/MC
								2.70 ± 1.40	High kinematics	
[37]	Germany	Relevant	n.r.	≥2000	Mobile	– | 1	n.a.	10.7 ± 0.59	Superior, burnishing	mg/MC
									Inferior, abrasive wear	
					Fixed	– | 1		7.51 ± 0.29	Superior, burnishing	mg/MC
									Inferior, creep and minimal wear	
[66]	Italy	n.r.	n.r.	≥2000	Mobile	– | 14	n.a.	4.35 ± 2.00	Metallic block	mg/MC
								2.25 ± 2.20	Synthetic femur	
					Fixed	– | 14		1.3 ± 1.1	Metallic block	mg/MC
								3.4 ± 1.4	Synthetic femur	

Abbreviations: C.I., confidence interval; cem, cemented; COIs, conflicts of interest; FU, follow‐up; LoE, level of evidence; Mc, million cycles; n.a., not applicable; n.r., not reported; uncem, uncemented.

**Table 2b:** Characteristics of single‐arm studies that report on wear rate for fixed‐ and mobile‐bearing designs.

Author; year	Country	Declarations		Inclusion period	Bearing design	Patients | knees	FU (years)	Wear rate	Comment	Unit
		Funding	COIs							
Retrospective (LoE IV single series; *n* = 2)										
[44]	UK	relevant	relevant	≥2000	Mobile (3rd gen)		14.1	60 ± 42		µm/year
					Mobile (1050 machined)		12.1	76 ± 32		µm/year
					Mobile (1050 moulded)		10	57 ± 30		µm/year
[30]	UK	Irrelevant	Relevant	<2000	Mobile (1st gen)		>17.2	0.070 (95% CI: 0.038–0.102)		mm/year
					Mobile (2nd gen)		>17.2	0.022 (95% CI: 0.017–0.027)		mm/year
Knee simulator (LoE V non‐comparative; *n *= 2)										
[59]	Germany	None	Relevant	≥2000	Fixed	– | 3	n.a.	3.0 ± 0.7	ISO 14243‐2:2009	mg/MC
								11.7 ± 3.0	Highly demanding	
[22]	Germany	n.r.	n.r.	≥2000	Fixed (AP)	– | 6	n.a.	8.6 ± 2.17	ISO 14243‐1:2002E	mm^3^/MC
					Fixed (CFR‐PEEK PAN)	– | 6		5.1 ± 2.29	ISO 14243‐1:2002E	mm^3^/MC
					Fixed (CFR‐PEEK PITCH)	– | 6		5.2 ± 6.92	ISO 14243‐1:2002E	mm^3^/MC
In silico (LoE V; *n* = 1)										
[36]	Korea	None	n.r.	≥2000	Mobile (anatomy mimetic, UHMWPE)	– | 1	n.a.	8.3	ISO 14243‐3	mm^3^/MC
					Mobile (increased conformity, UHMWPE)	– | 1		10.9	ISO 14243‐3	mm^3^/MC
					Mobile (anatomy mimetic, X‐UHMWPE)	– | 1		4.1	ISO 14243‐3	mm^3^/MC
					Mobile (increased conformity, X‐UHMWPE)	– | 1		5.3	ISO 14243‐3	mm^3^/MC
Conference abstract (LoE V non‐comparative; *n* = 1)										
[54]	UK	n.r.	n.r.	<2000	Mobile (1st gen)		22.3	0.072 ± 0.028		mm/year
					Mobile (2nd gen)		19.5	0.028 ± 0.019		mm/year

Abbreviations: AP, all‐polyethylene; C.I., confidence interval; CFR‐PEEK, carbon fibre‐reinforced poly‐ether‐ether‐ketone; COIs, conflicts of interest; FU, follow‐up; gen, generation; LoE, level of evidence; Mc, million cycles; n.a., not applicable; n.r., not reported; PAN, polyacrylonitrile; PITCH, discontinuous pitch fibres; UHMWPE, ultra‐high molecular weight polyethylene; X‐UHMWPE, cross‐linked ultra‐high molecular weight polyethylene.

**Figure 2:**
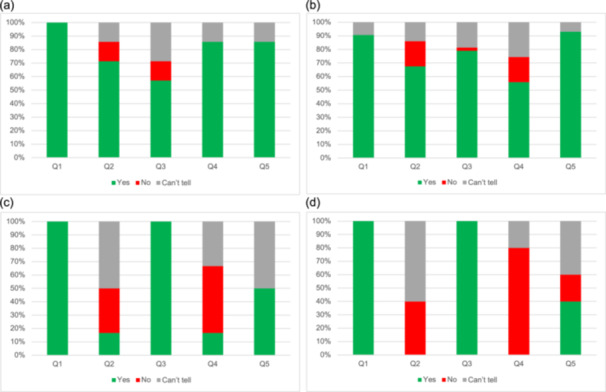
Quality assessment of included studies using the mixed methods appraisal tool. (a) Comparative studies on the incidence of wear. (b) Single‐arm studies on the incidence of wear. (c) Comparative studies on wear rates. (d) Single‐arm studies on wear rates.

The risk of bias varied across the four subgroups. Among the comparative studies reporting on the incidence of wear, all studies used a relevant sampling strategy, five (71%) had representative samples [28, 51, 61, 67, 72], four (57%) used appropriate measurements [13, 51, 60, 61], six (86%) showed a low risk of nonresponse bias and employed appropriate statistical analyses [28, 51, 60, 61, 67, 72] (Figure 2a). For the single‐arm studies, 41 (91%) used relevant sampling strategies [3, 5, 10, 12, 16, 17, 19, 21, 23, 26, 27, 29, 31, 34, 38, 40, 42, 45, 47, 55, 56, 58, 62, 64, 65, 68, 71, 73, 75], 29 (64%) had representative samples [3, 5, 6, 8, 9, 12, 20, 26, 27, 29, 38, 42, 53, 56, 58, 62, 64, 65, 68, 71, 73, 75], 36 (80%) used appropriate measurements [4, 5, 9, 10, 12, 16, 17, 19, 21, 23, 26, 27, 29, 31, 34, 38, 41, 42, 45, 55, 56, 58, 62, 64, 65, 68, 71, 73, 75], 26 (58%) showed a low risk of nonresponse bias [3, 4, 7, 9, 16, 19, 21, 23, 27, 29, 31, 32, 34, 38, 41, 42, 55, 58, 62, 64, 65, 68, 69, 75] and 41 (91%) employed appropriate statistical analyses [3, 8, 10, 12, 16, 17, 19, 21, 23, 26, 27, 29, 31, 32, 34, 38, 42, 45, 53, 55, 56, 58, 62, 64, 65, 68, 71, 73, 75] (Figure 2b). In the comparative studies reporting on wear rate, all studies used relevant sampling strategies and appropriate measurements; however, only one (17%) study had a representative sample and a low risk of nonresponse bias [25], and three (50%) [25, 37, 48] employed appropriate statistical analyses (Figure 2c). Finally, among the single‐arm studies, all used relevant sampling strategies and appropriate measurements; none had representative samples or showed a low risk of nonresponse bias; and only two (40%) [22, 44] used appropriate statistical analyses (Figure 2d).

### Analysis of incidence of wear for implants manufactured before 2000

**Figure 3:**
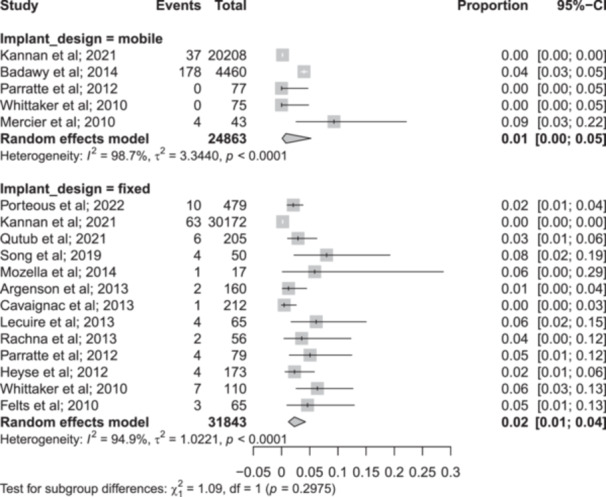
Analysis of the incidence of wear for implants manufactured before 2000.

There were no statistically significant differences (*p* = n.s.) in incidence of wear between mobile‐bearing (1%; *I* ^2^ = 98.7%) and fixed‐bearing designs (2%; *I* ^2^ = 96.0%) (Figure 3). For mobile‐bearing designs, the pooled estimate of incidence of wear increased to 2% and the heterogeneity reduced to 0% (Figure S1), while for fixed‐bearing designs, the pooled estimate of incidence of wear increased to 3% and the heterogeneity reduced to 72% when omitting Kannan et al. [28] (Figure S2).

### Analysis of incidence of wear for implants manufactured in or after 2000

**Figure 4:**
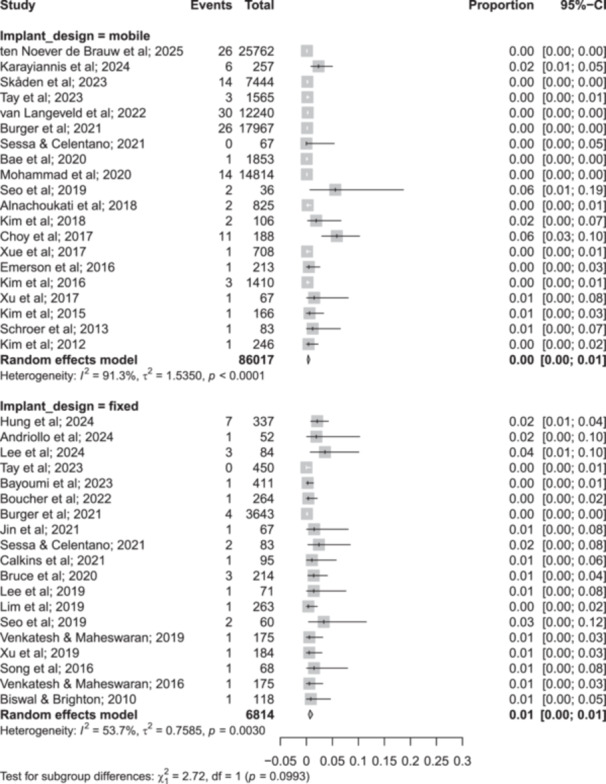
Analysis of the incidence of wear for implants manufactured in or after 2000.

There were no statistically significant differences (*p* = n.s.) in incidence of wear between mobile‐bearing (<1%; *p* = n.s.; *I* ^2^ = 91.3%) and fixed‐bearing designs (1%; *I* ^2^ = 53.7%) (Figure 4). For mobile‐bearing designs, the pooled estimate of incidence of wear and heterogeneity remained consistent (Figure S3), while for fixed‐bearing designs, the pooled estimate of incidence of wear also remained consistent, but the heterogeneity reduced to 0% when omitting Burger et al. [13] (Figure S4). For studies reporting on mobile bearing designs, a statistically significant funnel plot asymmetry was observed (Peters' test, *p* < 0.001), indicating a potential risk of publication bias or small‐study effects. Asymmetry was characterised by a high degree of heterogeneity, with eight studies falling outside the pseudo‐95% confidence limits: four reporting incidence rates lower than the pooled estimate and four reporting higher rates (Figure S5). Likewise, for studies reporting on fixed‐bearing designs, a statistically significant funnel plot asymmetry was observed (Peters' test, *p* = 0.007), with a disproportionate number of smaller studies reporting higher logit‐transformed proportions of implant wear. Four studies fell outside the pseudo‐95% confidence intervals, of which three were on the right side of the plot, suggesting that small‐study effects or publication bias may be influencing the pooled estimate (Figure S6).

**Figure 5:**
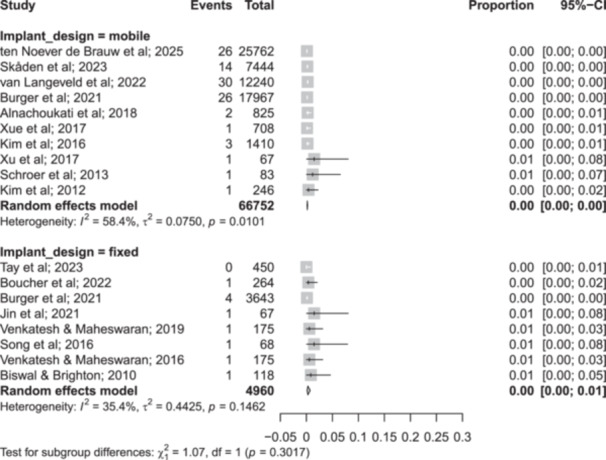
Analysis of the incidence of wear for implants manufactured in or after 2000 with <10 years of follow‐up.

For studies with <10 years of follow‐up, there were no statistically significant differences (*p* = n.s.) in incidence of wear between mobile‐bearing (0%; *I* ^2^ = 58.4%) and fixed‐bearing designs (0%; *I* ^2^ = 35.4%) (Figure 5). For mobile‐bearing designs, the pooled estimate of incidence of wear remained consistent and the heterogeneity reduced to 33% when omitting Ten Noever de Brauw et al. [68] (Figure S7), while for fixed‐bearing designs, the pooled estimate of incidence of wear remained consistent and the heterogeneity reduced to 0% when omitting Burger et al. [13] (Figure S8).

**Figure 6:**
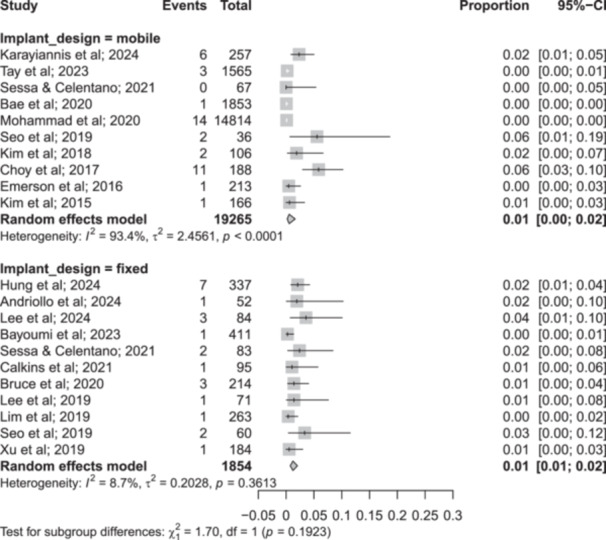
Analysis of the incidence of wear for implants manufactured in or after 2000 with >10 years of follow‐up.

For studies with >10 years of follow‐up, there were no statistically significant differences (*p* = n.s.) in incidence of wear between mobile‐bearing (2%; *I* ^2^ = 93.4%) and fixed‐bearing designs (1%; *I* ^2^ = 8.7%) (Figure 6). For mobile‐bearing designs, the pooled estimate of incidence of wear remained consistent, and the heterogeneity reduced to 84% when omitting Tay et al. [67] (Figure S9). For fixed‐bearing designs, the pooled estimate of incidence of wear increased to 2% when omitting Bayoumi et al. [8] and heterogeneity reduced to 0% when omitting either Lee et al. [40], Lim et al. [41] or Bayoumi et al. [8] (Figure S10). For studies reporting on mobile bearing designs, visual inspection of the funnel plot revealed substantial dispersion of studies, with five outliers falling outside the pseudo‐95% confidence limits (two on the left and three on the right; Figure S11). This distribution suggests that the asymmetry is driven more by high between‐study heterogeneity than by a systematic suppression of specific results (Peters' test, *p* = 0.0824). For studies on fixed‐bearing design, visual inspection of the funnel plot suggested a degree of asymmetry (Figure S12); however, Peters' regression test did not confirm statistically significant funnel plot asymmetry (*p* = 0.177), and the observed distribution likely reflects substantial between‐study heterogeneity rather than definitive publication bias.

### Analysis of wear rate

**Figure 7:**
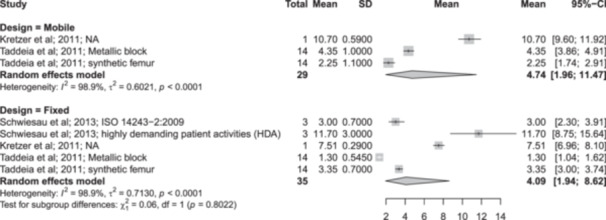
Analysis of the gravitational wear rate.

For studies measuring wear rate in mg/MC, there were no statistically significant differences (*p* = n.s.) between mobile‐bearing (4.74 mg/MC; *I* ^2^ = 98.9%) and fixed‐bearing designs (4.09 mg/MC; *I* ^2^ = 98.9%) (Figure 7). For mobile‐bearing designs, the pooled mean wear rate increased to 6.83 mg/MC when omitting Taddei et al. (synthetic femur) [66], while heterogeneity remained consistent (Figure S13), while for fixed‐bearing designs, the pooled mean wear rate increased to 5.44 mg/MC when omitting Taddei et al. (metallic block) [66], with heterogeneity remaining consistent (Figure S14).

**Figure 8:**
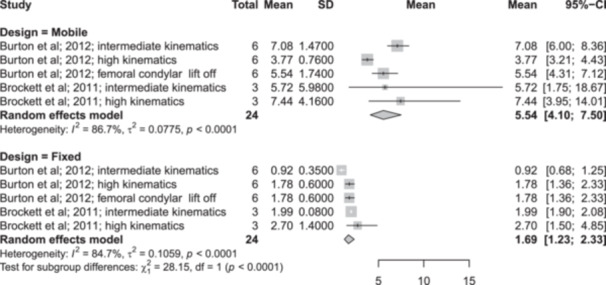
Analysis of the volumetric wear rate.

For studies measuring volumetric wear rate (mm^3^/MC), there were statistically significant differences (*p* < 0.0001) between mobile‐bearing (5.54 mm^3^/MC [*n* = 24 knees; *I* ^2^ = 86.7%]) and fixed‐bearing designs (1.69 mm^3^/MC [*n* = 24 knees; *I* ^2^ = 84.7%]) (Figure 8). For mobile‐bearing designs, the pooled mean wear rate increased to 6.48 mm^3^/MC, and the heterogeneity reduced to 0% when omitting Burton et al. (high kinematics) [15] (Figure S15), while for fixed‐bearing designs, the pooled mean wear rate remained consistent, but heterogeneity reduced to 0% when omitting Burton et al. (intermediate kinematics) [15] (Figure S16).

**Figure 9:**
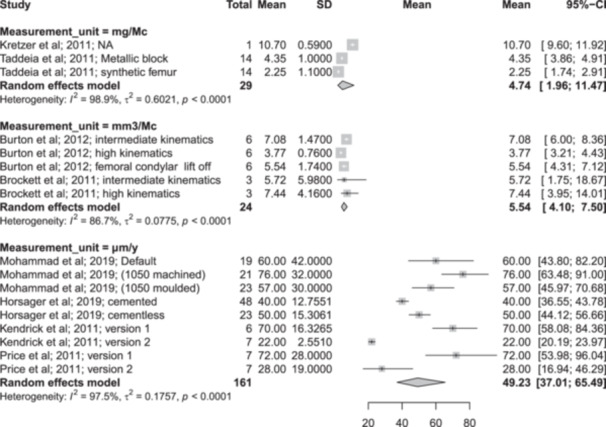
Analysis of the linear wear rate.

Linear wear rate (µm/year) was reported only for mobile‐bearing designs, with a pooled mean wear rate of 49.23 µm/year (*n* = 161 knees; *I* ^2^ = 97.5%) (Figure 9). The pooled mean wear rate increased to 55.46 µm/year when omitting Kendrick et al. (version 2) [30], with heterogeneity remaining consistent (Figure S17).

## DISCUSSION

The most important findings of this systematic review and meta‐analysis were that, although there was no significant difference in the clinical incidence of revision for wear between fixed‐ and mobile‐bearing constructs, fixed‐bearing constructs demonstrated a statistically significant advantage for in vitro volumetric wear rates. These findings add to the ongoing debate regarding the reported advantages of mobile‐bearing constructs and suggest that, in terms of linear or volumetric wear, both designs offer at least comparable long‐term durability.

The results align with several previous investigations reporting similar wear characteristics between fixed and mobile bearings in UKA [1, 43, 63], despite the differing biomechanical rationales underlying these designs. Mobile‐bearing implants were developed to reduce contact stresses by allowing greater conformity and self‐alignment, theoretically lowering wear. The results show volumetric wear was greater with mobile‐bearing implants, which is at odds with the reported theoretical advantage of this implant design. Volumetric wear provides a more comprehensive measurement than linear wear alone, and this finding may be clinically relevant, given its direct relationship with the risk of osteolysis.

Interestingly, there was a slight reduction in the rate of wear with the mobile‐bearing design in the cohort of papers which examine implants used prior to the year 2000. This difference, however, was not statistically significant. Examining the implants following the year 2000, coinciding with the use of more modern, wear‐resistant polyethylene designs, there was no statistically significant difference between mobile‐ and fixed‐bearing designs. This analysis provides evidence to suggest that the previously reported reduction in wear with the mobile‐bearing design may no longer be present. The results of the present study demonstrate no statistically significant difference in wear between the two implant designs when accounting for the advent of fixed‐bearing designs incorporating improved polyethylene processing.

Analysis of the studies reporting on wear in mobile‐bearing designs post‐2000 revealed significant heterogeneity (*I* ^2^ = 91.3%), whereas the fixed‐bearing design cohort reported moderate heterogeneity (*I* ^2^ = 53.7%). A sensitivity analysis was also performed, which revealed that the observed mean wear rate is consistent across the included studies for both mobile and fixed‐bearing papers, with no single study exerting a disproportionate influence on the overall result, despite the observed heterogeneity. While there are many factors which can affect heterogeneity across the results of each study, the reduced variance observed in the fixed‐bearing cohort may reflect more predictable, consistent kinematic patterns compared to mobile‐bearing designs. Another possible explanation for this finding is that the mobile‐bearing cohort may encompass a wider range of patient‐specific factors that influence implant position, soft‐tissue balancing and gait dynamics, resulting in greater variability in wear outcomes. While the results show no difference in wear between the two designs, the increased variability with mobile‐bearing designs may have implications for predicting individual implant performance or estimating the long‐term risk of revision following UKA.

This study combines all current evidence published in the literature and demonstrates that there is a comparable wear rate between the two designs. The continued use of both fixed and mobile‐bearing UKA is therefore supported, emphasising that factors other than wear, such as surgical preference, implant‐related complications or registry revision rates, may be more relevant when selecting a bearing design. Furthermore, the lower heterogeneity observed in the fixed‐bearing group may indicate more predictable wear behaviour, which may be advantageous when modelling longevity or counselling patients about expected outcomes.

There are, however, limitations of the study to consider. The sample size and specific patient cohorts of individual papers included in the study may limit generalisability, along with the fact that a greater number of mobile‐bearing designs in comparison to fixed are available in the published literature and therefore included in the study for analysis. Subtle differences in surgical technique, alignment, or postoperative activity levels could influence reported wear patterns. The cut‐off date of 2000 for the transition from conventional to cross‐linked polyethylene does not account for gradual and varied adoption by manufacturers and across regions. Although wear assessment techniques used in this study are robust, all wear‐measurement methodologies carry inherent assumptions that may affect precision. Furthermore, assumptions were made on reported revision rates due to wear, particularly with clinical and registry‐based studies. Revision surgery is influenced by many other factors aside from implant wear, and so this may under‐ or overestimate the actual occurrence of revision directly due to implant‐related wear. Pooled estimates for incidences of wear were based on raw proportions without adjusting for person‐time exposure. Not all included studies stated person‐time exposure or provided Kaplan‐Meier survival curves with wear as endpoint. The authors decided against estimating person‐time exposure from aggregate data since long‐term cohorts (>10 years) could be susceptible to higher attrition and loss to follow‐up rates compared to short‐term cohorts (<10 years). Failing to properly account for dropouts over a long period inflates the total exposure time, thereby artificially lowering the calculated incidence of revision. The temporal subgroup analysis mitigates exposure bias without introducing estimation errors. It should be noted that pooled analyses had substantial heterogeneity, and that this variability limits the certainty with which pooled estimates can be interpreted. Data from laboratory and in silico studies that reported on linear and volumetric wear rates should be interpreted with caution, as sample sizes were small, and testing protocols were different, with no minimum number of test cycles specified by the authors.

Future research should explore whether the observed differences in variability translate into meaningful differences in long‐term survivorship or complication rates. Larger, multicentre analyses or prospective registry‐based studies may help to clarify whether patient‐ or surgeon‐level predictors drive the heterogeneity in mobile‐bearing implants. Moreover, advanced imaging or computational modelling could provide more detailed insights into the mechanical reasons behind the variability in wear behaviour, such as the increased rate of volumetric wear seen in the mobile‐bearing group.

## CONCLUSION

This comprehensive review of all available data demonstrates that fixed‐ and mobile‐bearing medial UKAs show no difference in the clinical incidence of revision for wear but reveals a statistically significant advantage for fixed‐bearing medial UKAs regarding volumetric wear. Fixed‐bearing implants show more uniform wear patterns, suggesting potentially more predictable long‐term performance.

## AUTHOR CONTRIBUTIONS

**Oliver G. B. Dixon**: Investigation; methodology; validation; writing—original draft. **Emily M. London**: Investigation; methodology; validation; writing—review and editing. **Ahmed Elsaifi**: Methodology; writing—original draft. **Jacobus H. Müller**: Statistical analysis; writing—original draft. **Nick J. London**: Conceptualisation; validation; writing—review and editing.

## FUNDING INFORMATION

The authors have no funding to report.

## CONFLICT OF INTEREST STATEMENT

Jacobus H. Müller editorial boards for *KSSTA* and *BMC Musculoskeletal Disorders*. Nick J. London declares a consultancy contract for teaching and development with Zimmer Biomet and royalties from Zimmer Biomet. The other authors declare no conflicts of interest.

## ETHICS STATEMENT

This was a systematic review of published literature, and no approval was necessary.

## Supporting information

Supporting File 1

Supporting File 2

Supporting File 3

Supporting File 4

Supporting File 5

Supporting File 6

Supporting File 7

Supporting File 8

Supporting File 9

Supporting File 10

Supporting File 11

Supporting File 12

Supporting File 13

Supporting File 14

Supporting File 15

Supporting File 16

Supporting File 17

Supporting File 18

## Data Availability

Upon reasonable request, the corresponding author can provide access to the data used for all analyses.
