# Supplementary figures and images for: ‘Wear advantage’ of mobile‐bearing unicompartmental knee arthroplasty is a myth: Higher volumetric wear without reduced revision rates compared to the fixed‐bearing design: A systematic review and meta‐analysis
Source: J Exp Orthop. 2026 Jul 9;13(3):e70837. doi: 10.1002/jeo2.70837 (PMC13348665; doi:10.1002/jeo2.70837)

## Sorted by Proportion

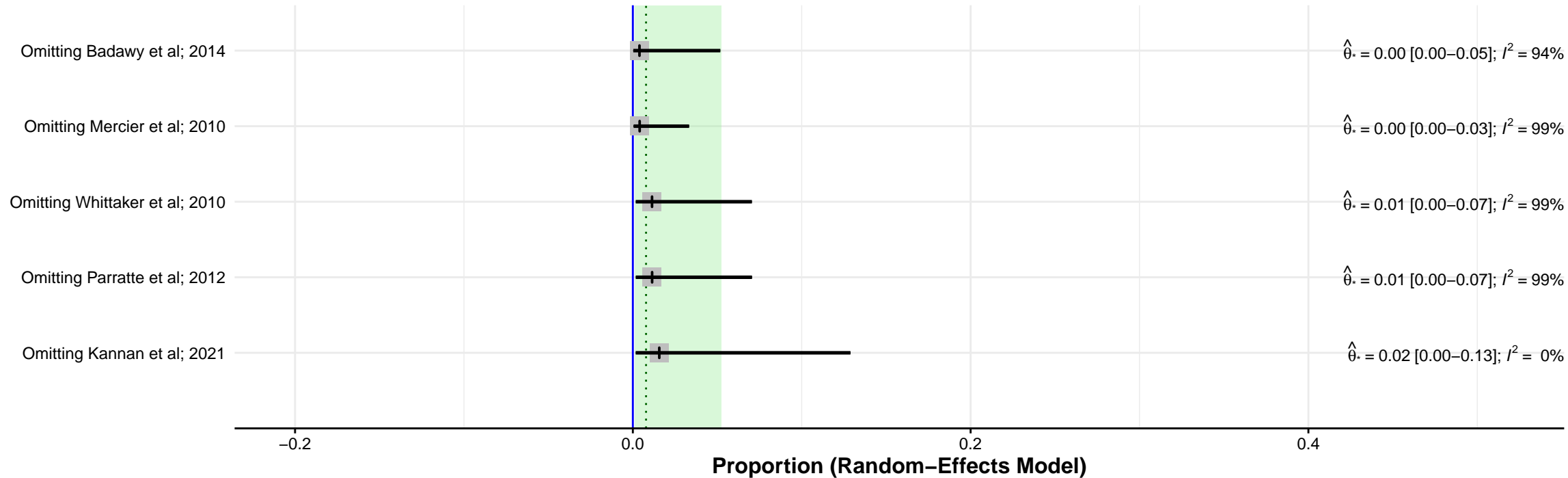

Supplement: Supplementary file 1 — Supporting File 1 [file JEO2-13-e70837-s002.pdf]

# Sorted by Proportion

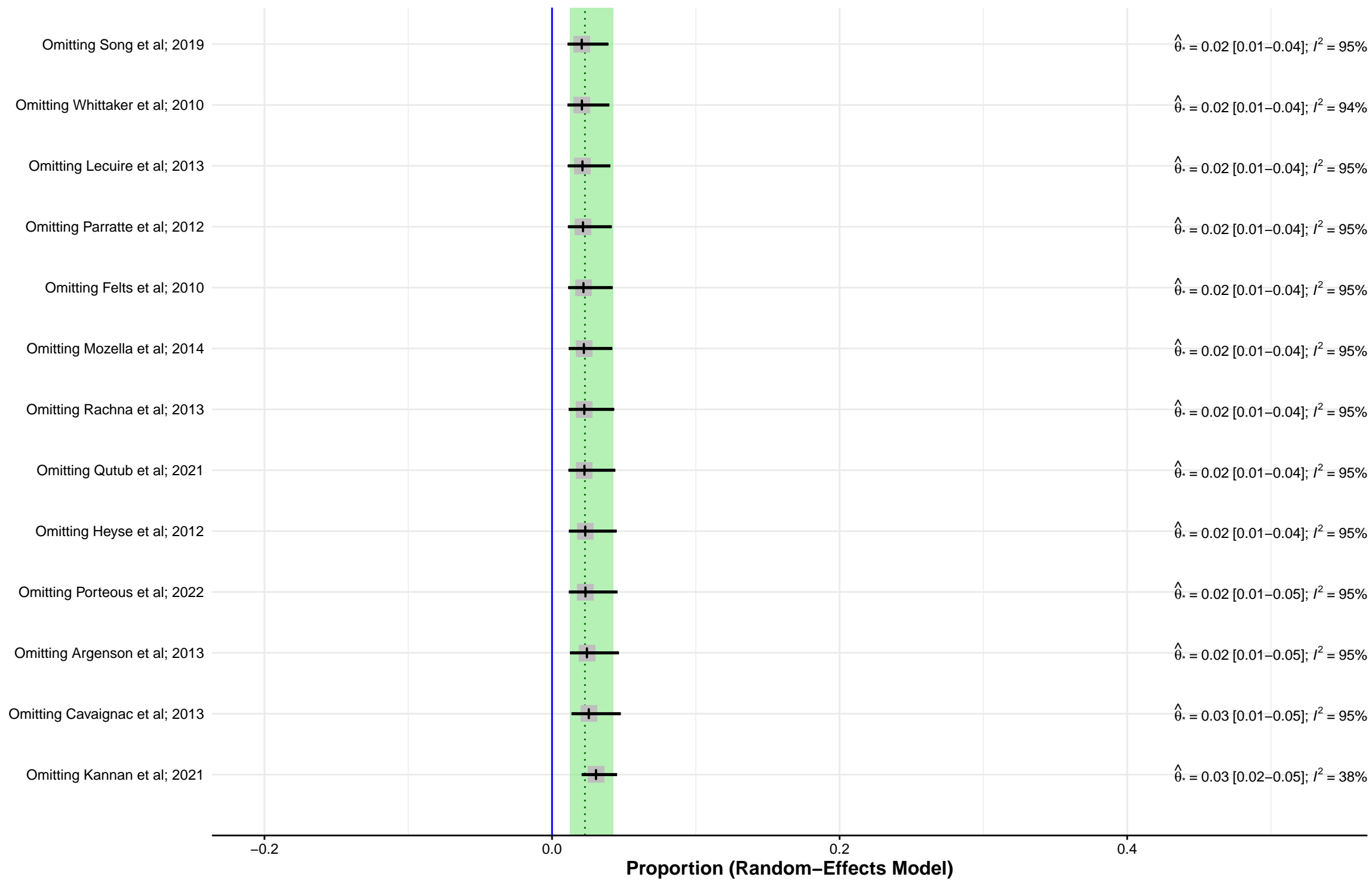

Supplement: Supplementary file 2 — Supporting File 2 [file JEO2-13-e70837-s013.pdf]

# Sorted by Proportion

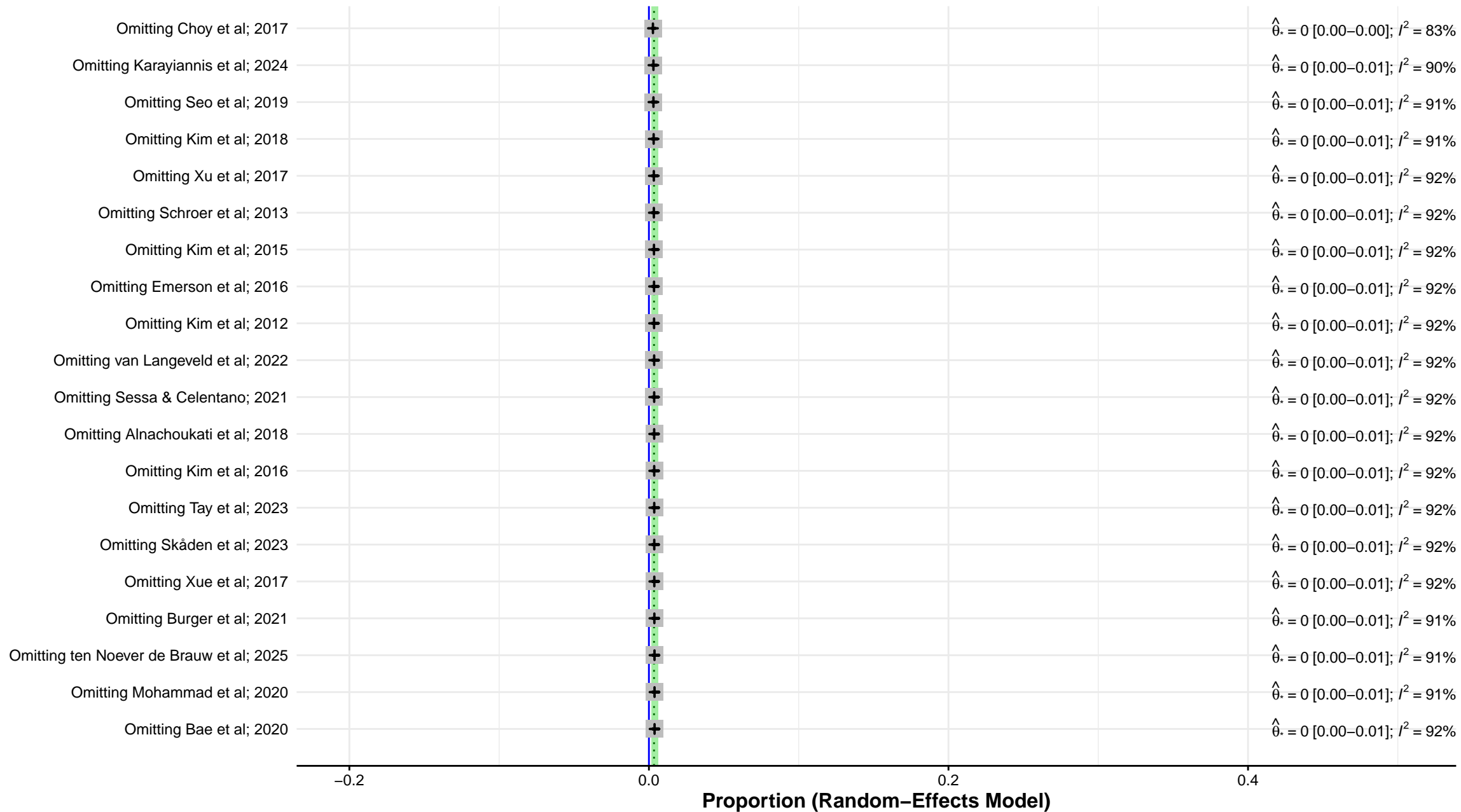

Supplement: Supplementary file 3 — Supporting File 3 [file JEO2-13-e70837-s004.pdf]

# Sorted by Proportion

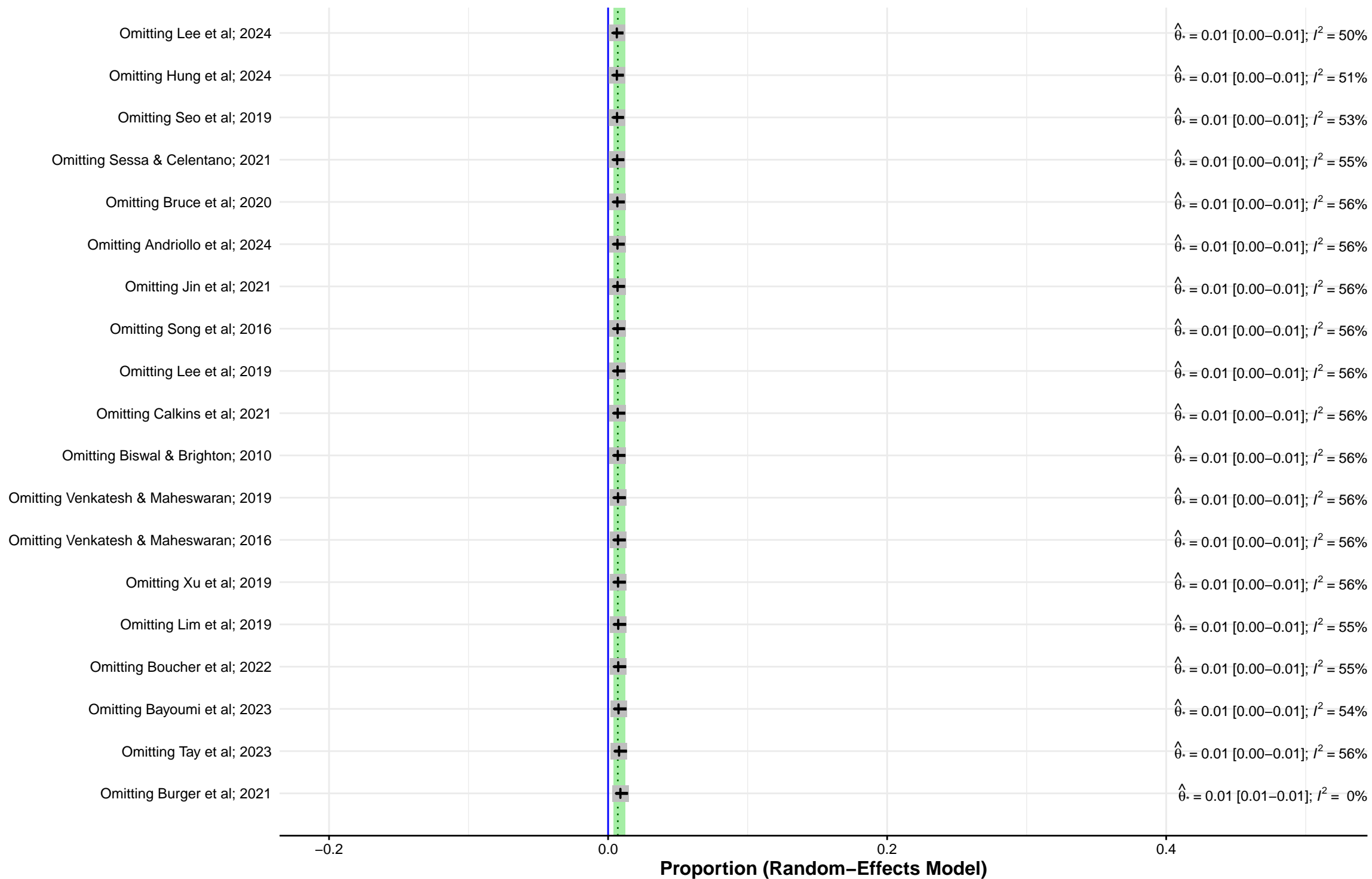

Supplement: Supplementary file 4 — Supporting File 4 [file JEO2-13-e70837-s003.pdf]

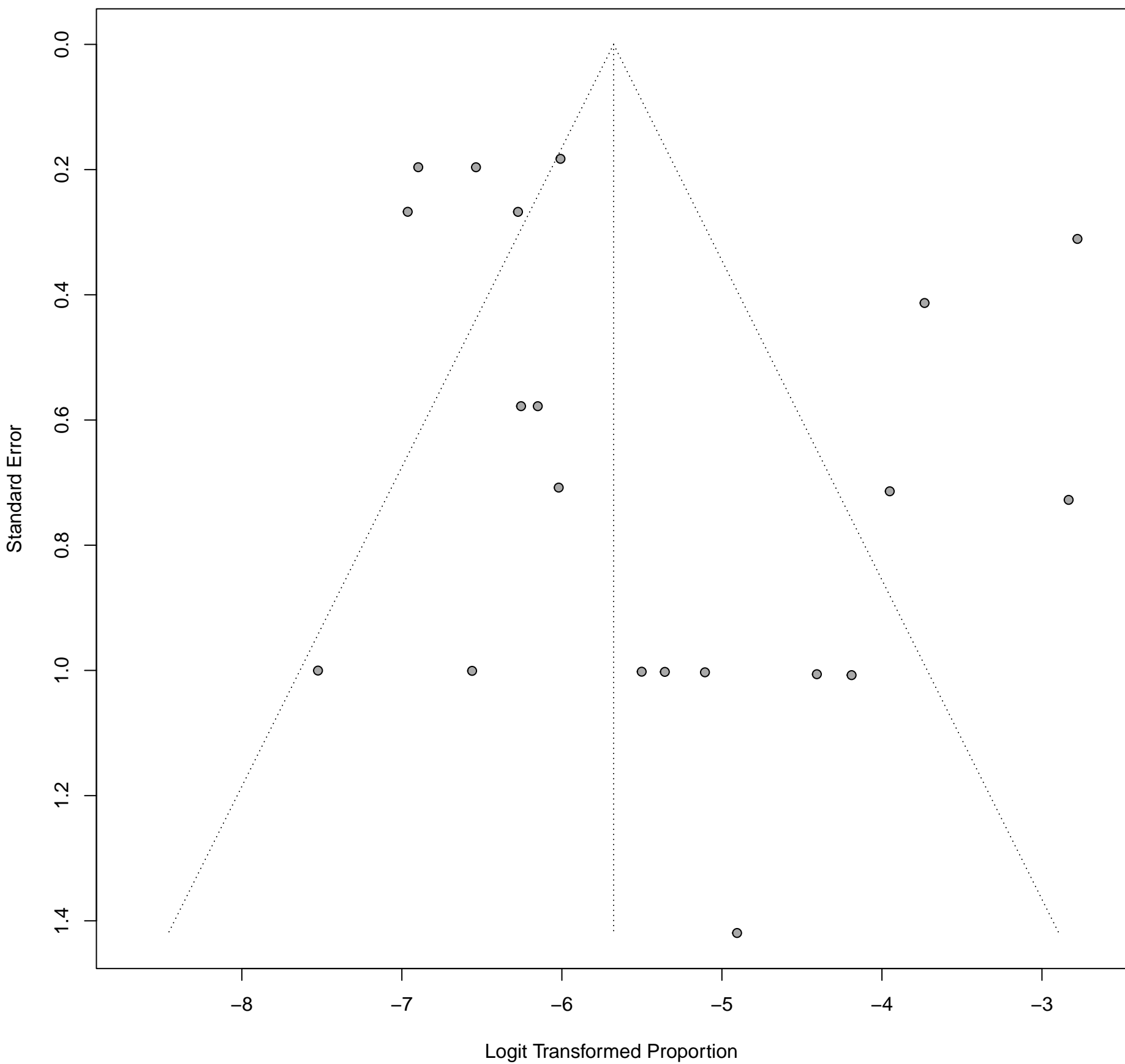

Supplement: Supplementary file 5 — Supporting File 5 [file JEO2-13-e70837-s010.pdf]

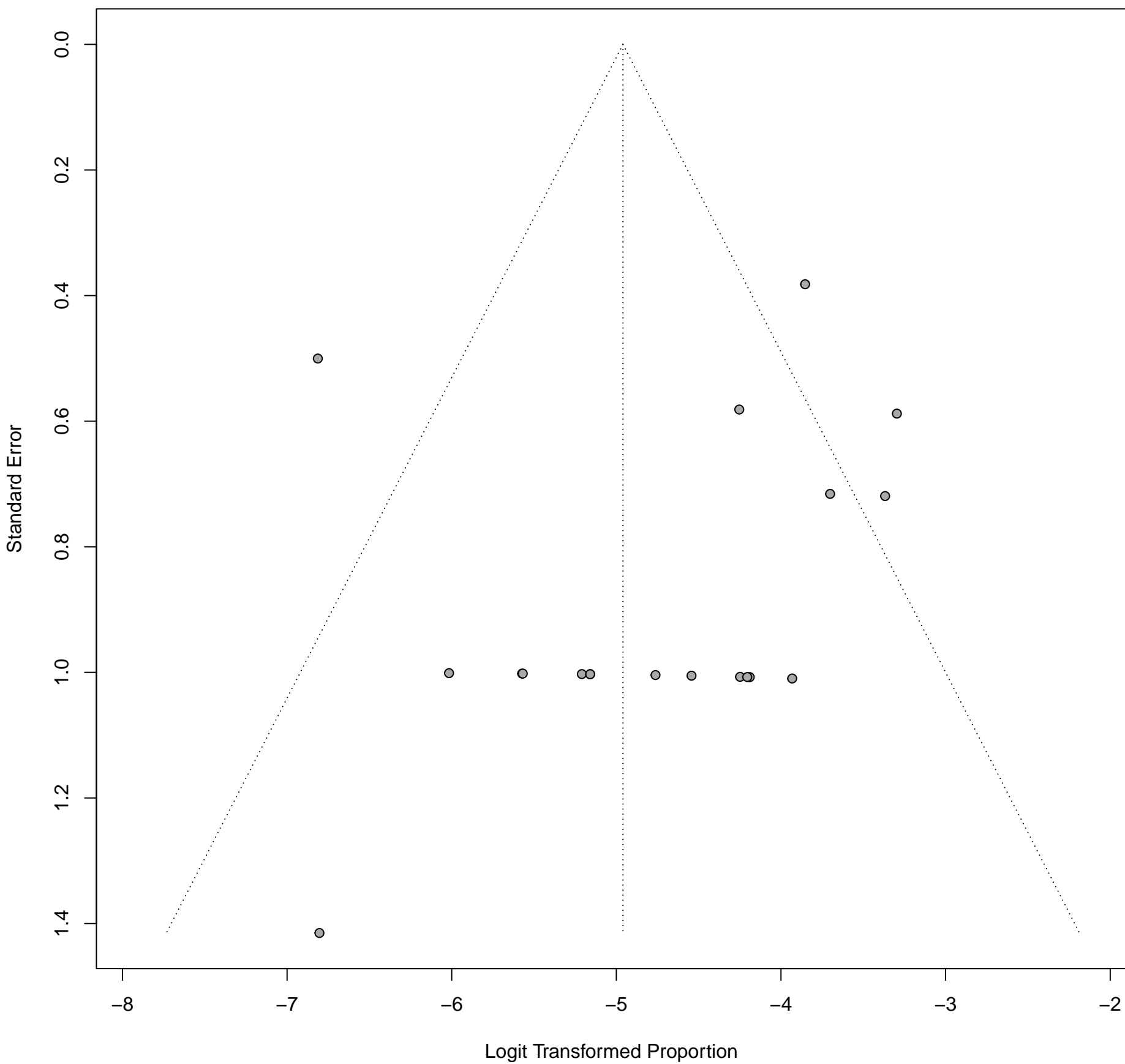

Supplement: Supplementary file 6 — Supporting File 6 [file JEO2-13-e70837-s009.pdf]

## Sorted by Proportion

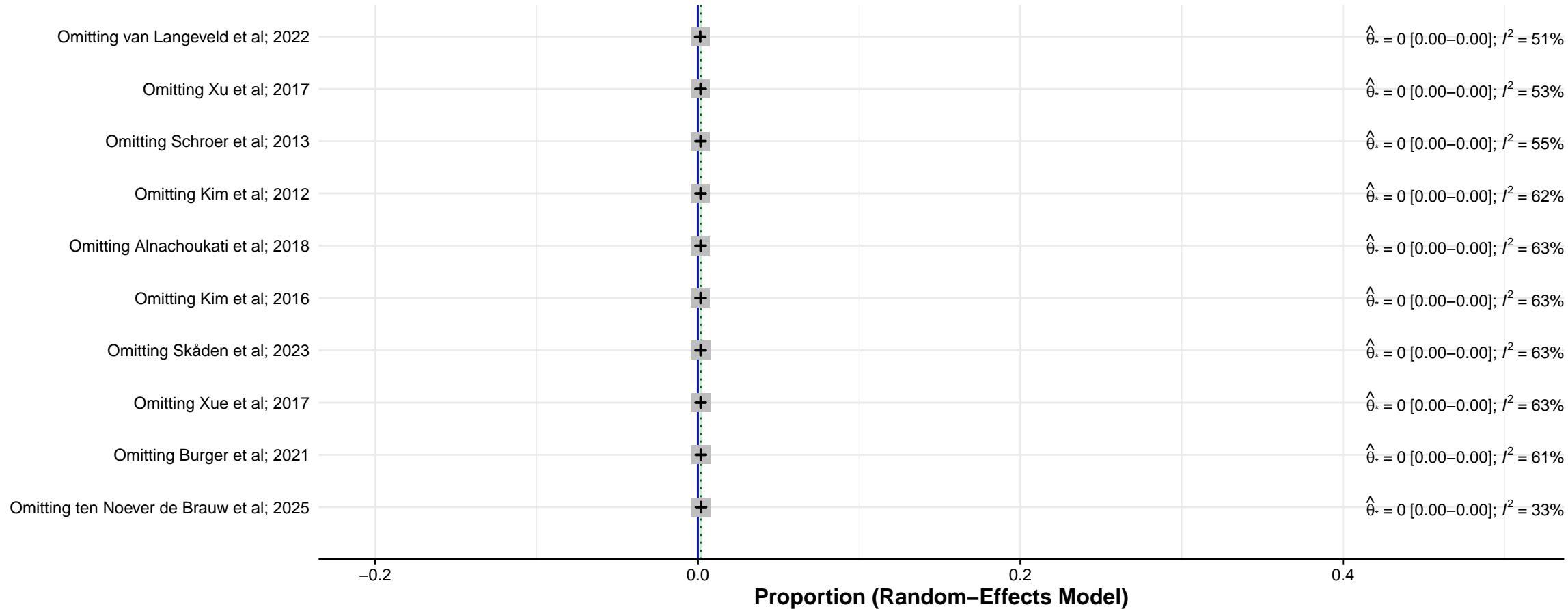

Supplement: Supplementary file 7 — Supporting File 7 [file JEO2-13-e70837-s008.pdf]

## Sorted by Proportion

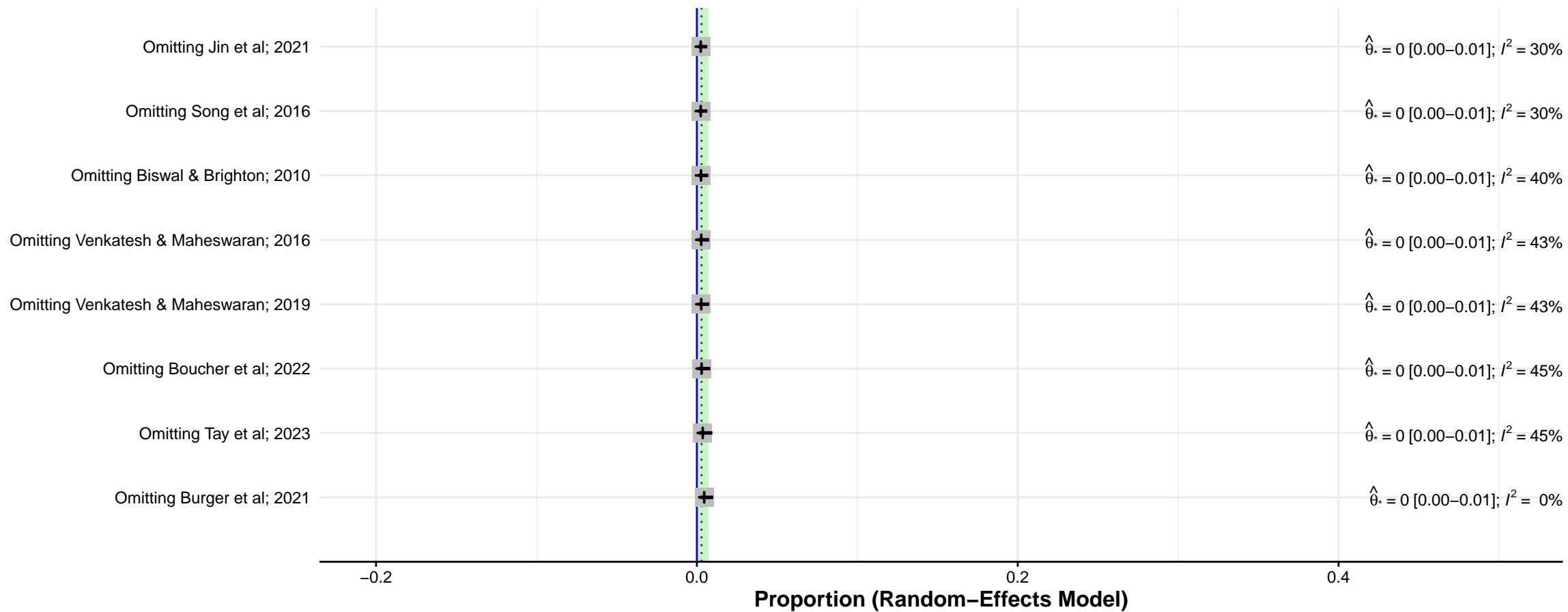

Supplement: Supplementary file 8 — Supporting File 8 [file JEO2-13-e70837-s011.pdf]

## Sorted by Proportion

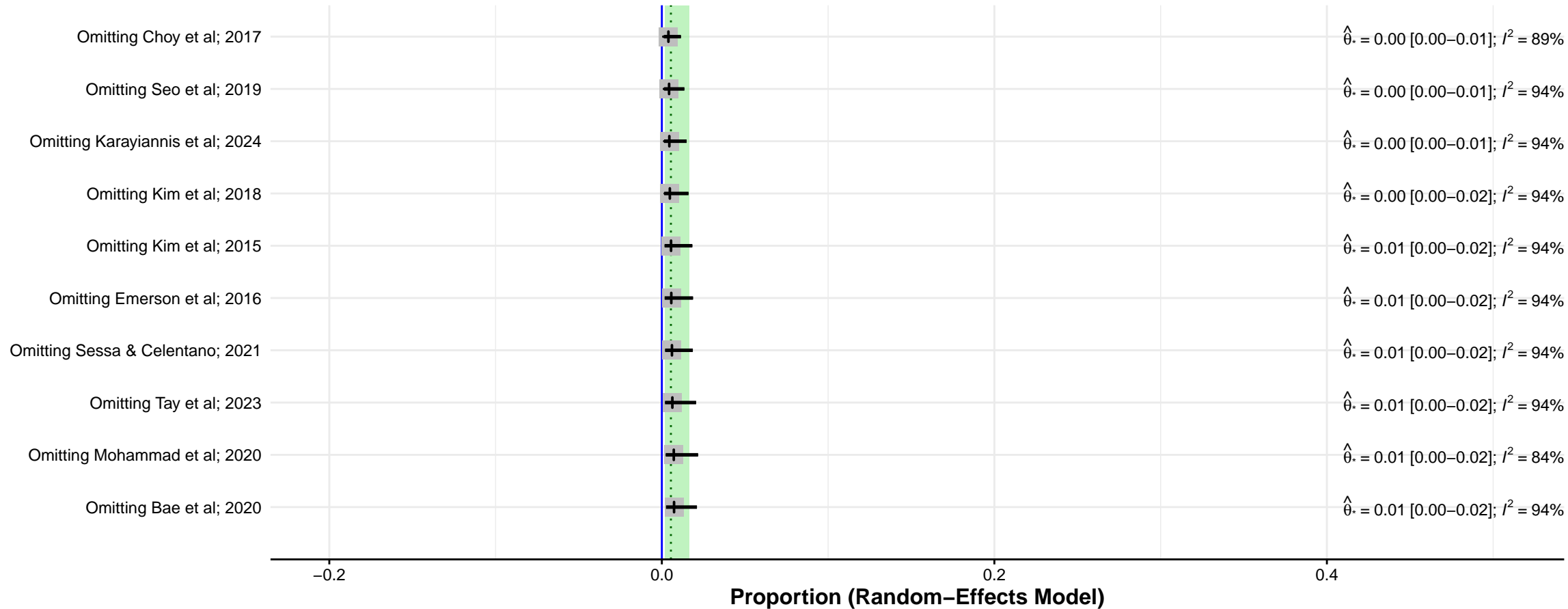

Supplement: Supplementary file 9 — Supporting File 9 [file JEO2-13-e70837-s015.pdf]

## Sorted by Proportion

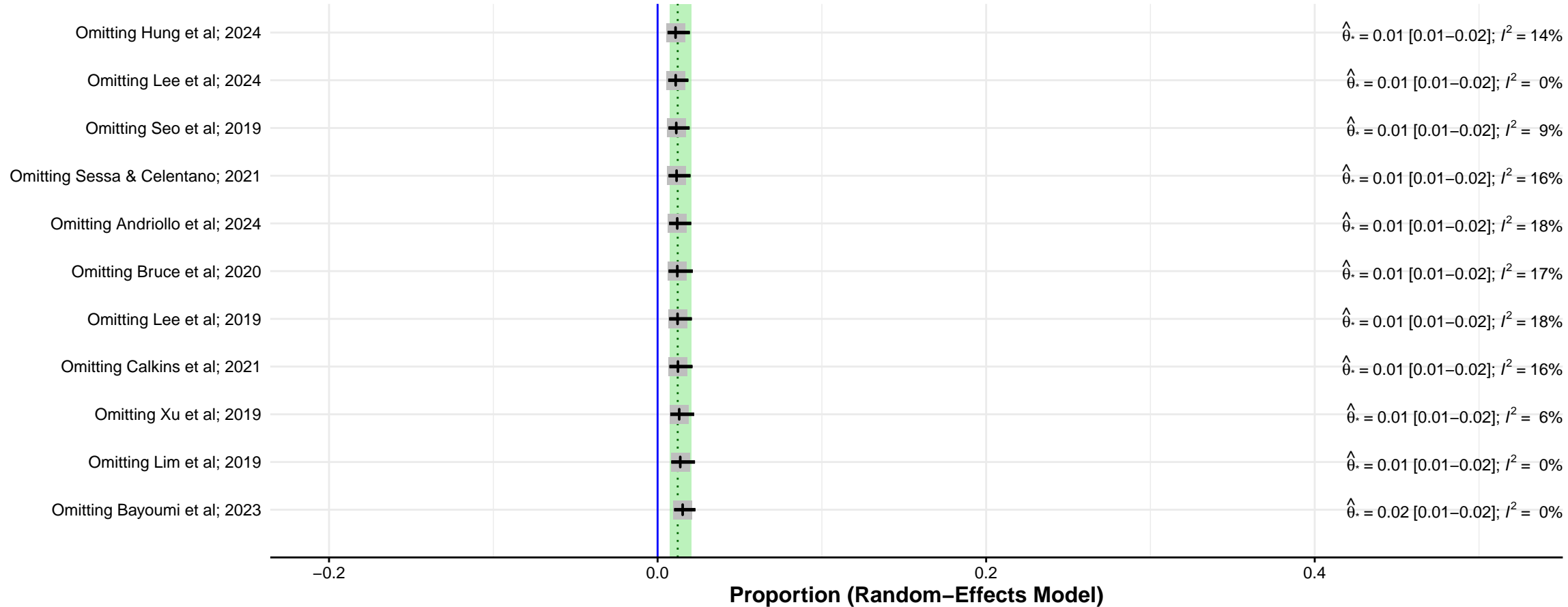

Supplement: Supplementary file 10 — Supporting File 10 [file JEO2-13-e70837-s007.pdf]

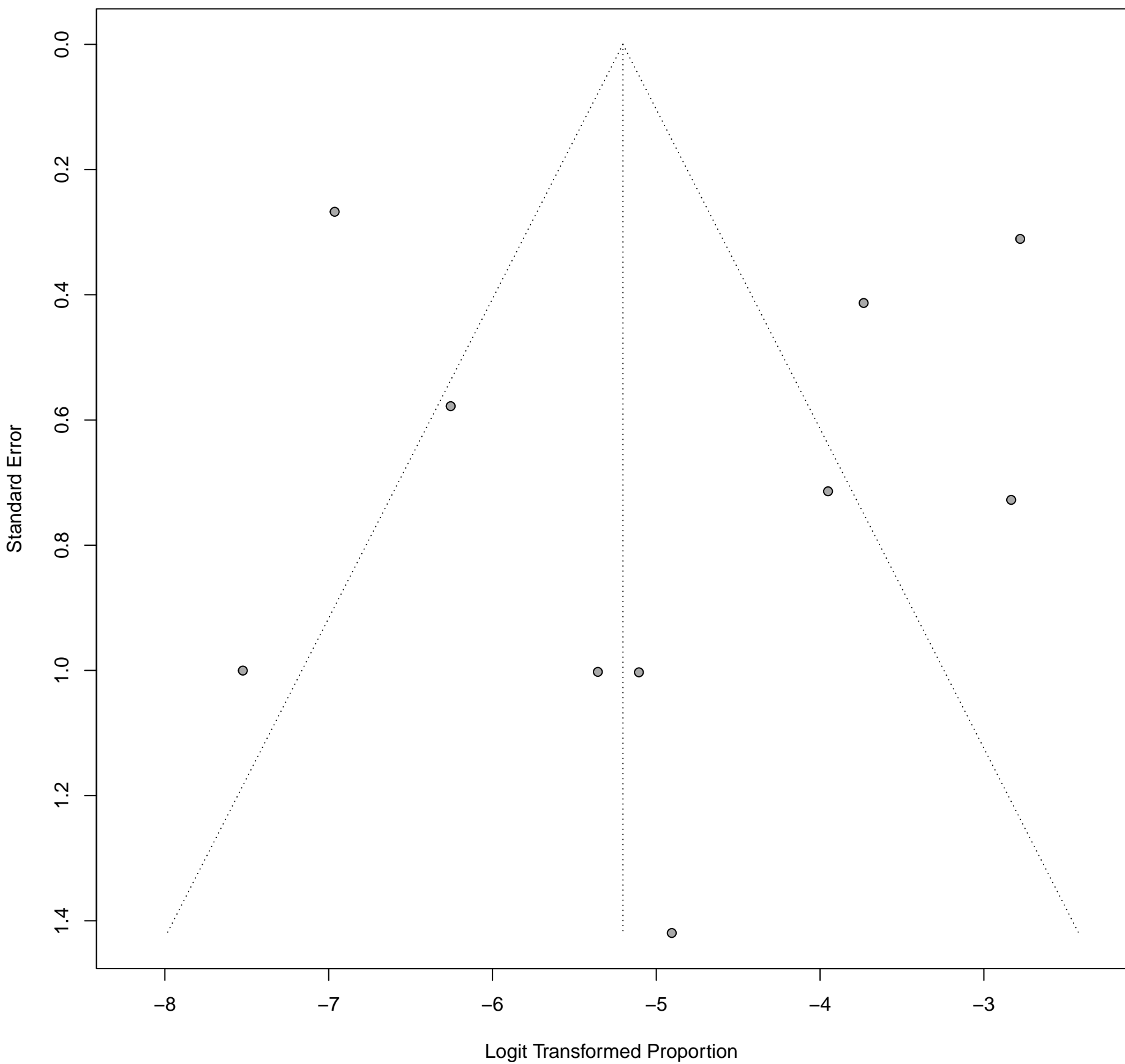

Supplement: Supplementary file 11 — Supporting File 11 [file JEO2-13-e70837-s017.pdf]

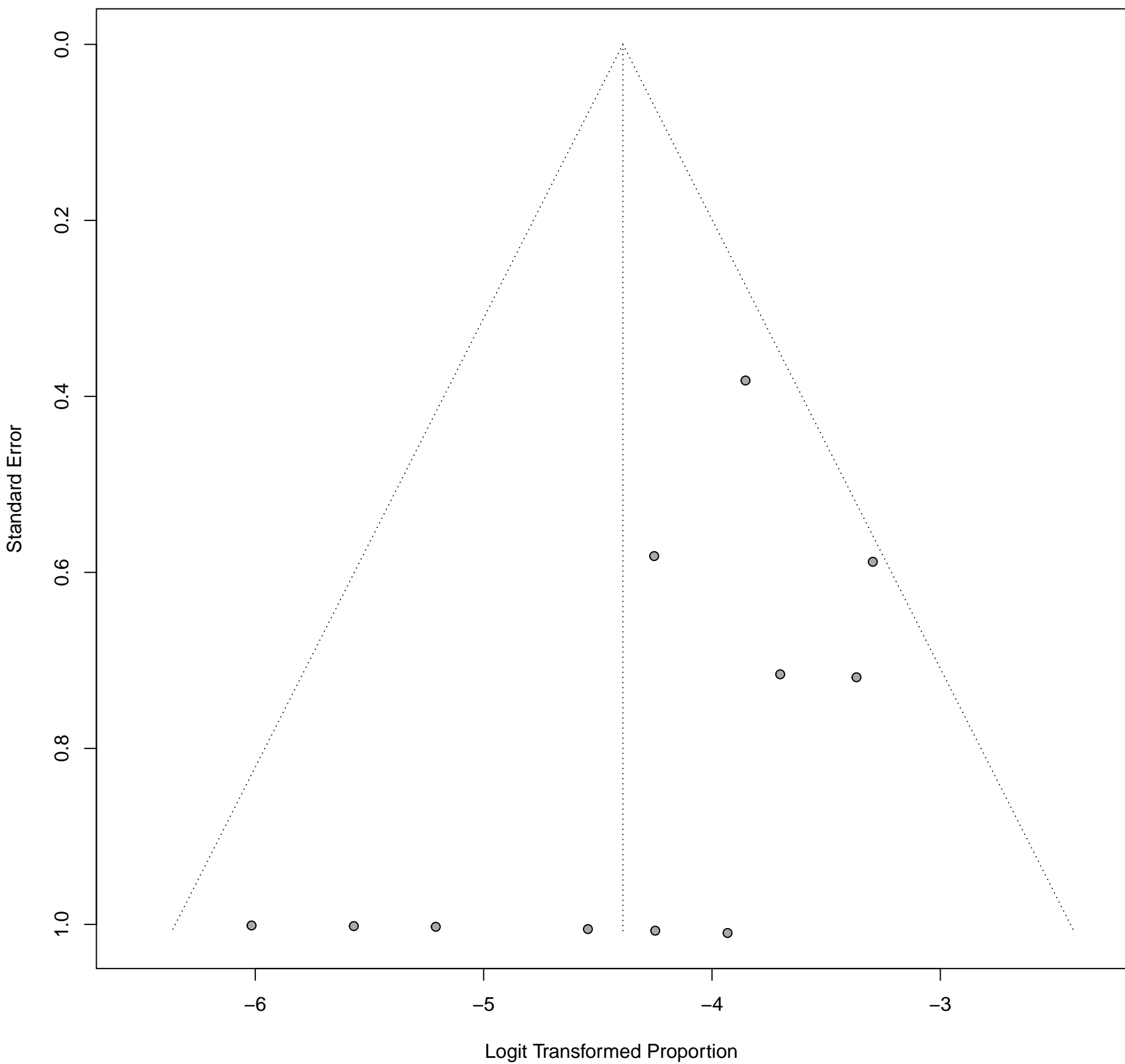

Supplement: Supplementary file 12 — Supporting File 12 [file JEO2-13-e70837-s016.pdf]

## Sorted by Effect Size

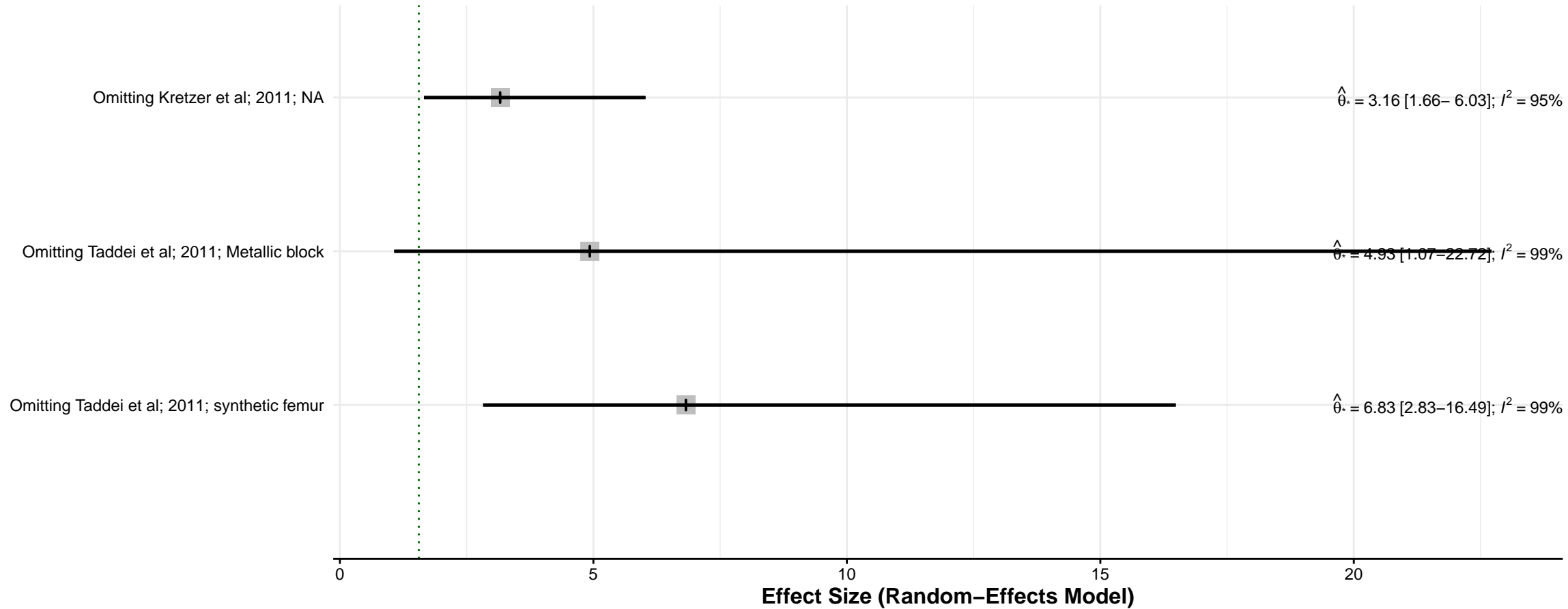

Supplement: Supplementary file 13 — Supporting File 13 [file JEO2-13-e70837-s005.pdf]

## Sorted by Effect Size

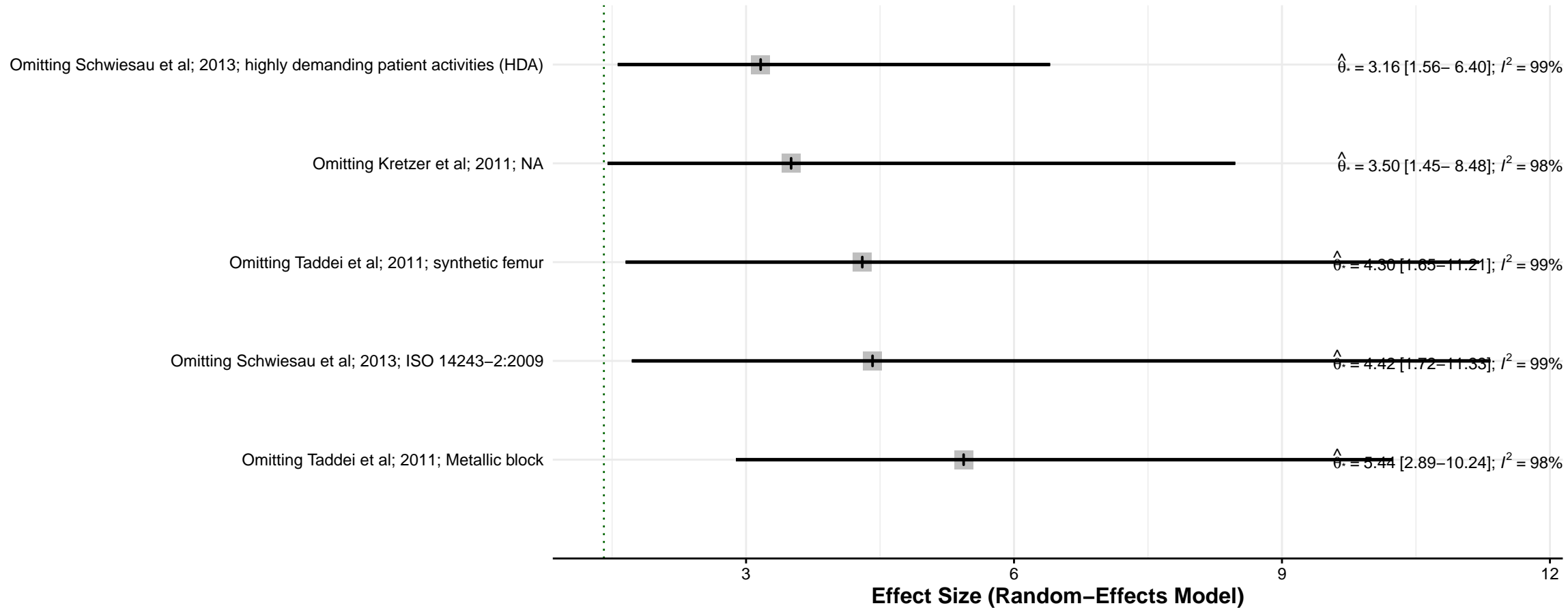

Supplement: Supplementary file 14 — Supporting File 14 [file JEO2-13-e70837-s018.pdf]

## Sorted by Effect Size

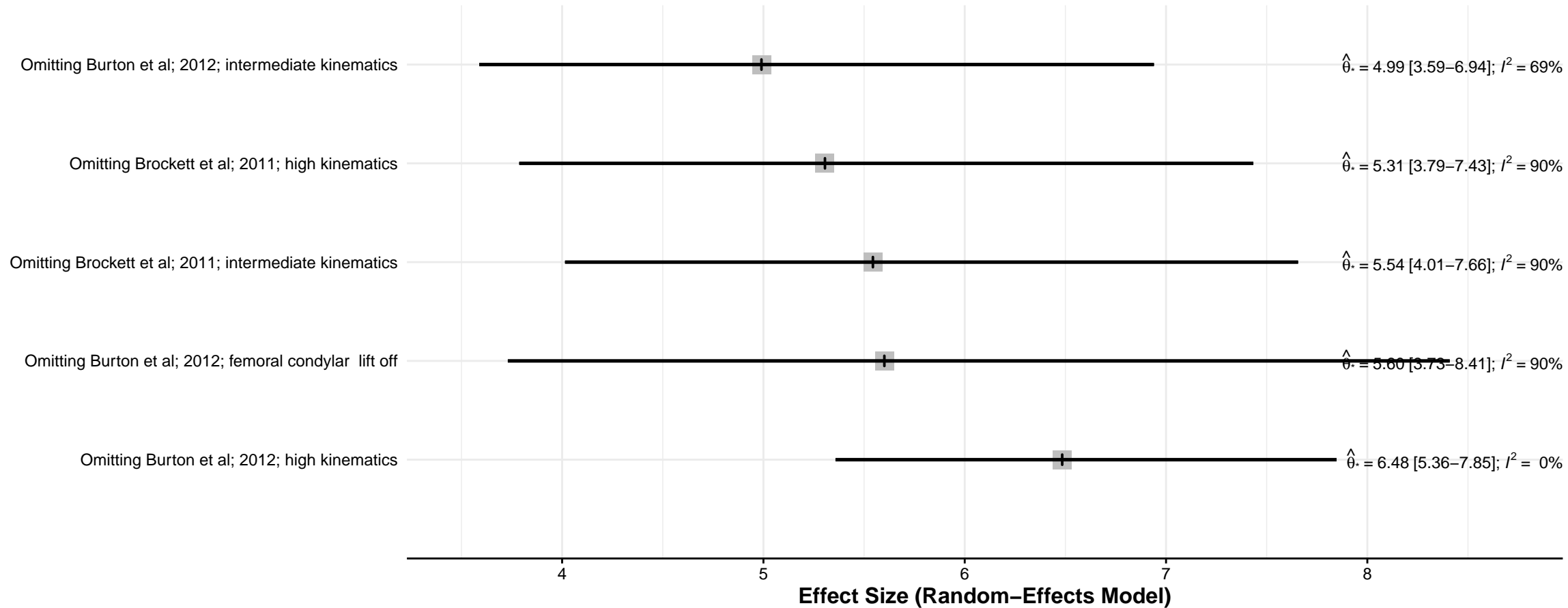

Supplement: Supplementary file 15 — Supporting File 15 [file JEO2-13-e70837-s001.pdf]

## Sorted by Effect Size

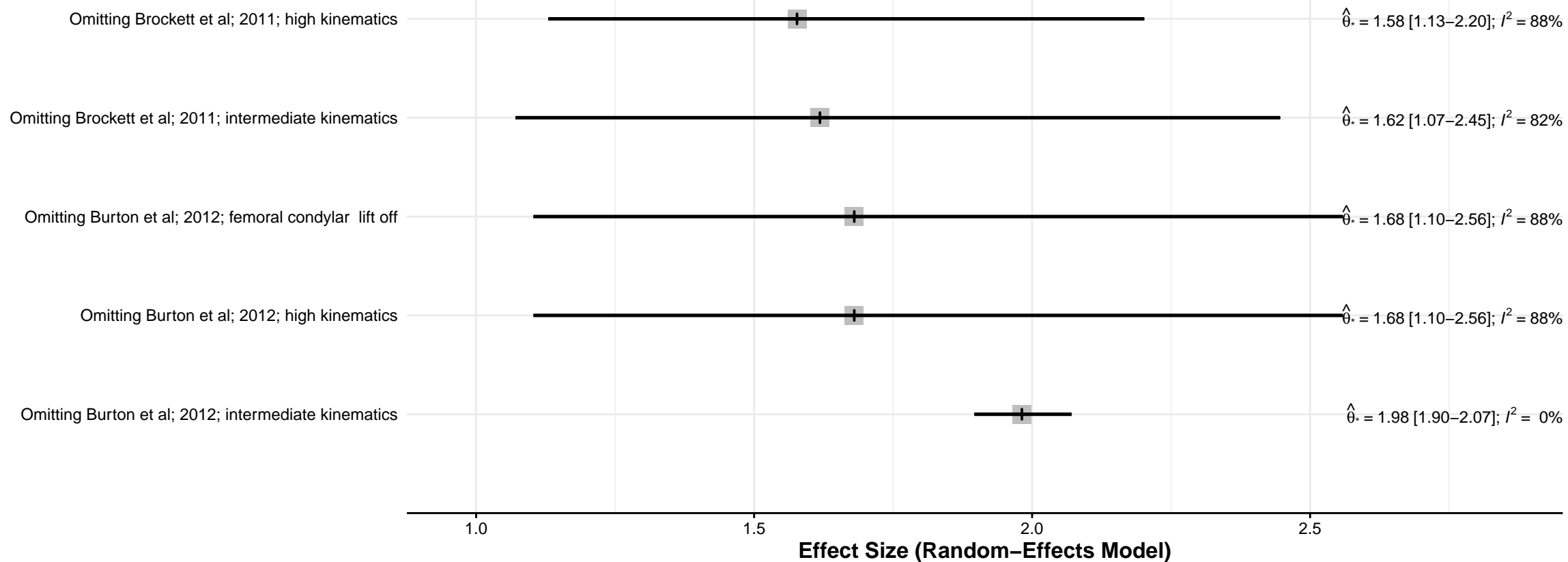

Supplement: Supplementary file 16 — Supporting File 16 [file JEO2-13-e70837-s012.pdf]

## Sorted by Effect Size

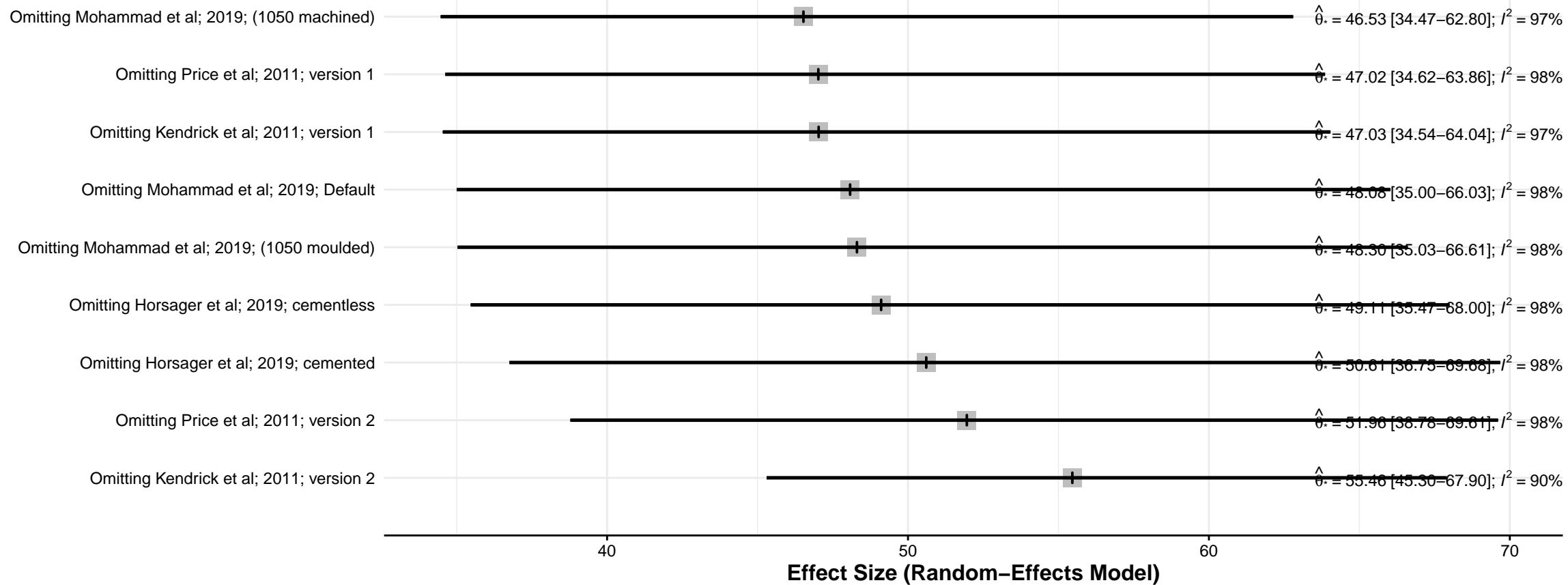

Supplement: Supplementary file 17 — Supporting File 17 [file JEO2-13-e70837-s014.pdf]
