# Supplementary material for: ‘Wear advantage’ of mobile‐bearing unicompartmental knee arthroplasty is a myth: Higher volumetric wear without reduced revision rates compared to the fixed‐bearing design: A systematic review and meta‐analysis
Source: J Exp Orthop. 2026 Jul 9;13(3):e70837. doi: 10.1002/jeo2.70837 (PMC13348665; doi:10.1002/jeo2.70837)
Supplement: Supplementary file 18 — Supporting File 18 [file JEO2-13-e70837-s006.docx]

Search strings

Pubmed:

| #1: **"unicompartmental knee arthroplasty" OR UKAOR"unicompartmental knee replacement" OR UKR OR"unicondylar knee arthroplasty" OR "unicondylar kneereplacement" OR"unicompartmental knee prosthesis" OR"unicompartmental knee implant" OR "unicondylar kneeprosthesis"** |
| --- |

#1: "unicompartmental knee arthroplasty" OR UKAOR"unicompartmental knee replacement" OR UKR OR"unicondylar knee arthroplasty" OR "unicondylar kneereplacement" OR"unicompartmental knee prosthesis" OR"unicompartmental knee implant" OR "unicondylar kneeprosthesis"

#2: ("2009/12/31"[Date - Publication] : "3000"[Date -Publication])

#3: "Lateral unicompartmental knee"[Title] OR "Lateralunicondylar knee"[Title]

#4: "systematic review"[Title] OR meta-analysis[Title]OR"meta analysis"[Title] OR "literature review"[Title] OR"scoping review"[Title] OR "narrative review"[Title]

#5: #1 AND #2

#6: #3 OR #4

#7: #5 NOT #6

#8: "mobile bearing"[Title/Abstract:~2] OR "fix bearing"[Title/Abstract:~2] OR "fixed bearing"[Title/Abstract:~2]

#9: #7 AND #8

#10: survival[Title/Abstract] OR survivorship[Title/Abstract]OR revision[Title/Abstract] OR revised[Title/Abstract]

#11: #7 AND #10

#12: wear[Title/Abstract] OR degradation[Title/Abstract] ORdamage[Title/Abstract] OR breakdown[Title/Abstract] ORfatigue[Title/Abstract]

#13: #7 AND #12

#14: #9 OR #11 OR #13

EMBASE:

S1: ("unicompartmental knee arthroplasty" OR UKA OR"unicompartmental knee replacement" OR UKR OR"unicondylarknee arthroplasty" OR "unicondylar knee replacement"OR "unicompartmental knee prosthesis" OR "unicompartmentalkneeimplant" OR "unicondylar knee prosthesis")

S2: ti("Lateral unicompartmental knee" OR "Lateral unicondylarknee")

S3: ti("systematic review" OR "meta-analysis" OR "meta analysis" OR"literature review" OR "scoping review" OR "narrativereview")

S4: (S2 OR S3)

S5: S1 NOT S4

S6: ab(mobile OR fix OR fixed) OR ab(bearing) OR ti(mobile OR fix ORfixed) OR ti(bearing)

S7: S5 AND S6

S8: ti(survival OR survivorship OR revision OR revised) OR ab(survivalOR survivorship OR revision OR revised)

S9: S5 AND S8

S10: ti(wear OR degradation OR damage OR breakdown OR fatigue) ORab(wear OR degradation OR damage OR breakdown OR fatigue)

S11: S5 AND S10

S12: S7 OR S9 OR S11
